# The B-MYB Transcriptional Network Guides Cell Cycle Progression and Fate Decisions to Sustain Self-Renewal and the Identity of Pluripotent Stem Cells

**DOI:** 10.1371/journal.pone.0042350

**Published:** 2012-08-24

**Authors:** Ming Zhan, Daniel R. Riordon, Bin Yan, Yelena S. Tarasova, Sarah Bruweleit, Kirill V. Tarasov, Ronald A. Li, Robert P. Wersto, Kenneth R. Boheler

**Affiliations:** 1 Bioinformatics Unit, National Institute on Aging, National Institutes of Health, Baltimore, Maryland, United States of America; 2 Molecular Cardiology and Stem Cell Unit, National Institute on Aging, National Institutes of Health, Baltimore, Maryland, United States of America; 3 Flow Cytometry Unit, National Institute on Aging, National Institutes of Health, Baltimore, Maryland, United States of America; 4 The Methodist Hospital Research Institute, Cornell University Weill Cornell Medical College, Houston, Texas, United States of America; 5 Department of Biology, Hong Kong Baptist University, Hong Kong, China; 6 Stem Cell and Regenerative Medicine Consortium, LKS Faculty of Medicine, University of Hong Kong, Hong Kong, China; National University of Singapore, Singapore

## Abstract

Embryonic stem cells (ESCs) are pluripotent and have unlimited self-renewal capacity. Although pluripotency and differentiation have been examined extensively, the mechanisms responsible for self-renewal are poorly understood and are believed to involve an unusual cell cycle, epigenetic regulators and pluripotency-promoting transcription factors. Here we show that B-MYB, a cell cycle regulated phosphoprotein and transcription factor critical to the formation of inner cell mass, is central to the transcriptional and co-regulatory networks that sustain normal cell cycle progression and self-renewal properties of ESCs. Phenotypically, B-MYB is robustly expressed in ESCs and induced pluripotent stem cells (iPSCs), and it is present predominantly in a hypo-phosphorylated state. Knockdown of B-MYB results in functional cell cycle abnormalities that involve S, G2 and M phases, and reduced expression of critical cell cycle regulators like *ccnb1* and *plk1*. By conducting gene expression profiling on control and B-MYB deficient cells, ChIP-chip experiments, and integrative computational analyses, we unraveled a highly complex B-MYB-mediated transcriptional network that guides ESC self-renewal. The network encompasses critical regulators of all cell cycle phases and epigenetic regulators, pluripotency transcription factors, and differentiation determinants. B-MYB along with E2F1 and c-MYC preferentially co-regulate cell cycle target genes. B-MYB also co-targets genes regulated by OCT4, SOX2 and NANOG that are significantly associated with stem cell differentiation, embryonic development, and epigenetic control. Moreover, loss of B-MYB leads to a breakdown of the transcriptional hierarchy present in ESCs. These results coupled with functional studies demonstrate that B-MYB not only controls and accelerates cell cycle progression in ESCs it contributes to fate decisions and maintenance of pluripotent stem cell identity.

## Introduction

All complex organisms contain stem cells with abilities to self-renew indefinitely and differentiate into one or many types of specialized cell types. These properties foster organismal development and promote cell replacement and organ repair in vivo. Pluripotent stem cells (PSCs), in particular, differentiate to all cell types of an embryo proper and may serve as an inexhaustible source of cell progeny useful for regenerative medicine. The best characterized and accepted standard for PSCs are embryonic stem cells (ESCs), which are derived from the inner cell mass (ICM) or epiblast of the mammalian blastocyst (reviewed in [1]). Experimentally derived PSCs, known as induced PSCs (iPSCs), can also be generated from somatic cells in vitro through forced expression of pluripotency-promoting transcription factors that include OCT4, SOX2, KLF4, c-MYC, LIN28 and NANOG [2], [3], [4]. Although experimentally-derived PSCs exhibit greater interline variation and differences in germ-line transmission than ESCs, iPSC lines may be more immunologically suited for regenerative medicine and disease modeling [5], [6], [7], [8], [9] [10], [11].

A defining feature of PSC biology is self-renewal, which can be aptly defined as the capacity to proliferate indefinitely while maintaining cell pluripotency [1], [12], [13], [14], [15], [16]. These two facets of self-renewal are believed to be intrinsically regulated through suppression of differentiation by polycomb group complexes (PcG), histone methylation, the presence of pluripotency transcription factors (TFs) [16], and to a unique ESC cell cycle [17], [18]. Importantly, the ability to self-renew is reduced or lost with cell commitment and differentiation, but it is re-acquired by somatic cells reprogrammed to iPSCs. Moreover, a direct link exists between a cell's proliferative ability and its capacity for reprogramming [19]; but, how cell cycle progression and self-renewal are established and maintained in PSCs is only partially understood. An improved comprehension of the mechanisms that control this process will contribute to our understanding of the biology of self-renewal and the reprogramming of somatic cells to iPSCs.

Attention to the proliferative component of self-renewal has focused principally on two key classes of regulatory molecules: a) cyclins and cyclin dependent kinases (Cdks), and b) proteins that regulate their assembly and activities [18], [20], [21], [22]. In ESCs, Cyclins D1, D3, E1, A2, and B1 are present, and except for Cyclin B, are stably expressed throughout the ESC cell cycle. Cdk activity is elevated and *cell cycle-independent*. Characteristically, Cdk inhibitory molecules are not expressed, members of the retinoblastoma (pRb) family are constitutively phosphorylated, and in the absence of these regulatory proteins, E2F target genes are thought to be actively transcribed throughout the ESC cell cycle [18], [20], [21], [22], [23]. Contributing to this regulation is the TF c-MYC [24], [25], which is part of a group of factors implicated in the reprogramming of somatic cells to iPSCs [2], [3]. C-MYC contributes to elevated levels of cyclins D2, E and A; whereas, diminished levels of c-MYC result in expression of regulatory cyclins and of cell cycle inhibitors p21^Cip1^ and p27^Kip1^ [18], [25]. While loss of c-MYC does not lead to a complete cessation of self-renewal processes, it causes reduced proliferation and differentiation defects [25].

Similar to c-MYC, the myeloblastosis oncogene-like 2 (MYBL2) TF, B-MYB, is critical for inner cell mass formation and ESC generation [26]. In somatic cells, the *mybl2* gene is normally up-regulated in late G1 and is thought to regulate progression into S phase. We recently demonstrated that B-MYB is also functionally implicated in proper progression through the S and G2/M cell cycle phases of ESCs, as loss of this TF causes replication fork defects and numerous flaws in mitosis, including severe mitotic spindle and centrosome defects, and aneuploidy [27], [28]. Although a few B-MYB regulated genes have been identified in somatic cells, most of the observed defects are mediated through currently undefined B-MYB target genes. Here, we have examined the role of B-MYB through genome-wide gene expression profiling, differential phosphorylation studies, and ChIP-chip experiments in ESCs and following B-MYB knockdown. These genome-wide analyses unraveled a complex B-MYB-mediated transcriptional network that regulates cell cycle progression, and significantly affects global transcriptional network connectivity, Cdk inhibitory molecule abundance, and key epigenetic modulators essential to stem cell identity. Integrated data analysis further demonstrate that signals responsible for regulating cell cycle progression and promoting self-renewal traits in ESCs converge through B-MYB.

## Results

### Knock-down, differential phosphorylation and functional assays of B-MYB in ESCs

**Figure 1:**
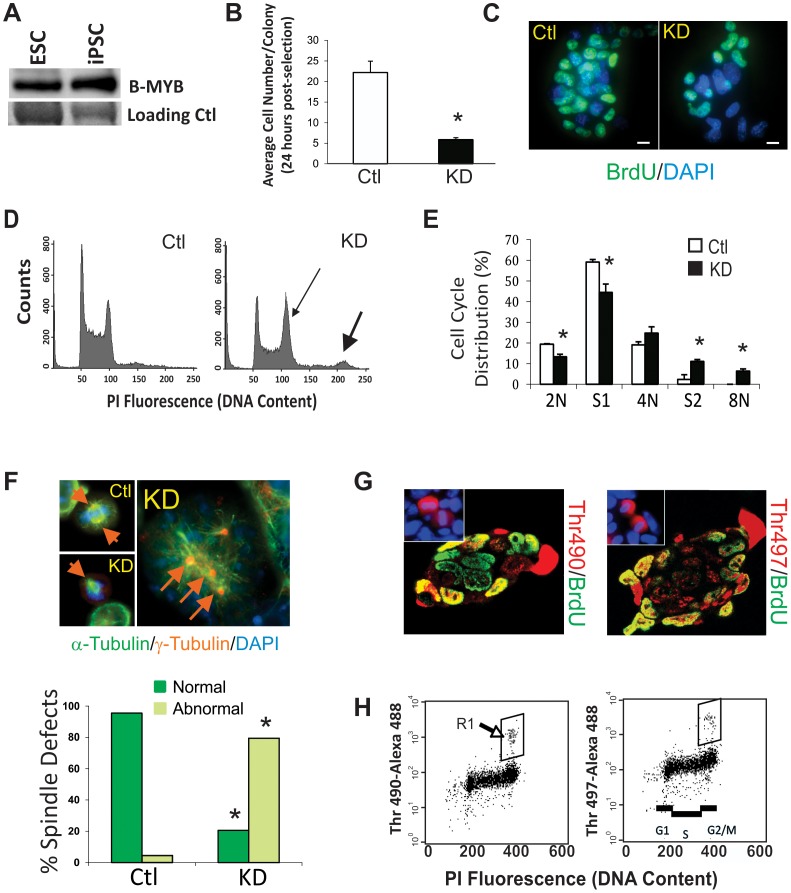
B-MYB function and phosphorylation in pluripotent stem cells. A) Western blot showing presence of B-MYB in ESCs (R1) and iPSCs (2D4). B) Graphic representation of the average number of cells present within individual ESC colonies (n = 40 colonies/group) 48 hours after nucleofection with control or targeting shRNA1 vectors to B-MYB. C) Typical image of pulsed BrdU incorporation in control and B-MYB deficient ESCs, showing decreased BrdU incorporation in B-Myb knockdown (KD) cells. D) DNA content measured by flow cytometry of non-synchronized fixed ES cells stained with propidium iodide after nucleofection with shRNA constructs. At 48 hours post-nucleofection, including 24 hours of selection with puromycin, a shift in the DNA content to a G2/M prevalent and aneuploid (8N) state (see arrows in inset) can be detected. E) Quantification of the cell cycle distributions following knockdown (KD) of B-MYB. The number of cells in S phase decreased concomitant to an increase in the number of cells in G2/M relative to controls and a significant increase in octoploidy (8N). F) Representative mitotic cells stained with DAPI (blue), α-tubulin (green) and γ-tubulin (red) are shown. In these experiments, mitotic spindle and centrosome defects are readily observed in cells lacking B-Myb. A quantitative assessment of spindle defects detected in B-Myb deficient cells (n = 3, 100 cells/group) is shown in the graph below. G) Images of immunostained ESC colonies with antibodies specific for phosphorylated forms of B-MYB. It is noteworthy that all mitotic cells show phosphorylation at Thr490 or Thr497. H) Flow cytometry analysis of mouse ESCs showing that phosphorylated forms of B-MYB are present only in the G2/M phases of the cell cycle (see boxed region). The number of cells present in the boxed regions correlates directly with the number of red mitotic cells shown in the inset of Figure 1G. Size marker = 15 µm. *, p<0.05. Data are expressed as mean ± standard deviation (SD).

As reported in our previous publication, B-MYB is highly abundant in ESCs, but here, we show for the first time that it is also highly expressed in iPSCs at levels similar to those seen in ESCs (Figure 1A). The functional significance of B-MYB in cell cycle control of PSCs was demonstrated through the use of short hairpin RNA (shRNA) constructs in transient knockdown experiments [27]. In this study, we principally employed shRNA1, which provided highly consistent functional results comparable to those found with either shRNA2 or shRNA5; however, these latter shRNAs were employed for validation experiments [27]. Consistent with our previous findings with shRNA1, 2 and 5, B-Myb RNA levels and B-MYB proteins levels were routinely decreased by >90% and by >70%, respectively (n = 8 for each condition). Knockdown of B-Myb resulted in small colonies consisting of fewer ESCs than that found in controls. These data are quantified in graphic form in Figure 1B. The number of cells within each colony that incorporated bromodeoxyuridine (BrdU) during S phase was also significantly reduced (p<0.05). Most BrdU negative cells in the knockdown experiments have slightly enlarged nuclei relative to controls, indicating some degree of cell differentiation. This finding is consistent with our previous report showing increased expression of differentiation markers CoupTF, Fgf5, Sox17, Cdx2 and Hand1 following knockdown of B-MYB [27] (Figure 1C). Knockdown of B-MYB also caused a significant increase in aneuploid cells with 8N chromosome content and an increased number of cells in G2/M with a corresponding decrease in G1 phase cells (Figure 1D), which we have quantified for the first time in Figure 1E. At a cellular level, a significant increase in monopolar and multipolar centrosomes with spindle defects was reconfirmed, showing that loss of B-MYB leads to profound cell cycle abnormalities (Figure 1F).

Post-translational phosphorylation of B-MYB does not account for the phenotypic changes observed in ESCs following knockdown. In somatic cells, hypo-phosphorylation is associated with increased B-MYB stability and activity [29], while site-specific phosphorylation in the conserved region and the negative regulatory domain of this protein results in altered transcriptional activity [30], [31], [32], [33], [34]. In ESCs, we show that B-MYB undergoes site specific phosphorylation in a cell cycle-dependent manner (Figure 1G–1H) that does not differ between control and knockdown conditions. In ESCs, phospho-Ser581, which is associated with transcriptional repression, was undetectable (not shown), but phosphorylated forms of Thr490 and Thr497, which are associated with transcriptional activation, were observed in ∼5–20% of the ESCs. These latter results are consistent with the post-translational modifications that we previously observed by two-dimensional gel electrophoresis [27]. In this study, phospho-Thr490 and phospho-Thr497 were only observed in the G2/M phases of the cell cycle (Figure 1H). B-MYB translocation to the extra-chromosomal space during late mitosis and cytokinesis was also preceded by phosphorylation, as mitotic cells were always phosphorylated (immunostaining in Figure 1G) and the numbers of mitotic cells correlated directly with the phosphorylated forms of B-MYB observed by flow cytometry (Figure 1H, boxed regions). Importantly, the number of cells with phospho-Thr490 or phospho-Thr497 B-MYB did not increase in proportion to the overall increase in cells present in the G2/M phases of the cell cycle following KD of B-MYB. This result suggests that B-MYB phosphorylation occurs primarily during late G2 or early M phase, since cells lacking B-MYB appear to be blocked in the early G2 phase of the cell cycle [27]. B-MYB is therefore hypo-phosphorylated in a majority of control ESCs; however, phosphorylation at sites Thr490 and Thr497 in late G2 or early M phase is oscillatory and intrinsic to the unusual cell cycle and self-renewal properties of ESCs.

**Figure 2:**
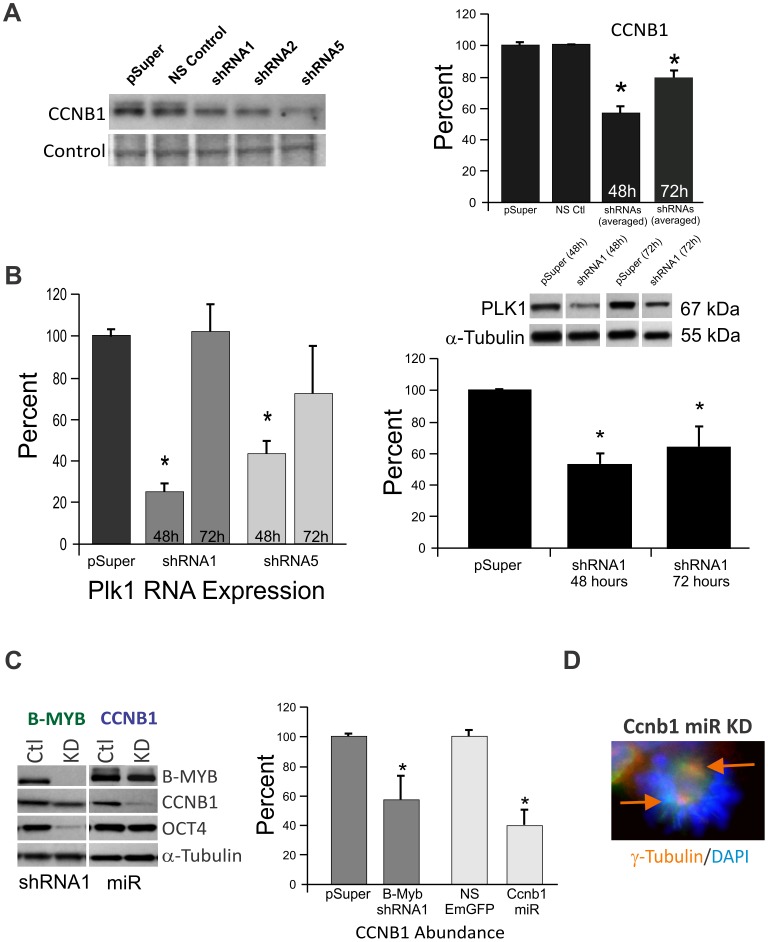
Examination of known somatic cell B-Myb target gene expression patterns following knockdown of B-Myb in ESCs. A) Western blot and graphic presentation of Cyclin B1 (Ccnb1) protein expression following knockdown of B-MYB by shRNAs 1, 2 and 5. B) RNA (left) and protein (right) analysis of the B-MYB target gene Polo-like kinase 1 (Plk1) following Bγ-Myb KD with shRNA1 and shRNA5. The effects of B-MYB KD on Plk1 RNA were transient; however, the loss of RNA led to a significant and sustained decrease in its protein abundance. C) Western blot and bar graph showing the abundance of selected proteins following knockdown of B-MYB and CCNB1 using shRNAs and microRNAs, respectively. D) Reduced expression of CCNB1 could not mimic the cell cycle related defects associated with B-MYB deficiency, such as monopolar or multipolar centrosomes as described in Figure 1. Ctl – control; KD – Knockdown; NS- non-silencing. *, p<0.05. Data are expressed as mean ± standard deviation (SD).

Knockdown of B-MYB causes a significant but transient decrease in the accumulation of known somatic cell B-MYB target gene products (Ccnb1, Cdca2 and Plk1) implicated in normal progression from G2 into M phases of the cell cycle. In Figure 2A, we show that the abundance of CCNB1 protein is reduced 48 hours after nucleofection regardless of the shRNA employed to knockdown B-MYB (shRNA1: 64%; shRNA2: 57%; and shRNA5: 41% relative to controls). CCNB1 protein levels remained significantly reduced 24 hours later; however, RNA expression levels returned toward control levels at this later time point (data not shown). Similar results were observed for Plk1. RNA levels were significantly reduced at 48 hours but not 72 hours post-nucleofection. In contrast, protein levels of PLK1 were significantly reduced at both time points (Figure 2B). Similar results were seen with CDCA2 (not shown), and we previously reported that Oct4 and Sox2 had comparable expression patterns. Based on these and subsequent analyses (see below), we conclude that transient but significant changes in RNA expression represented a common feature of this knockdown model system.

To determine if reduced expression of Ccnb1 could account for the B-MYB phenotype, as was the case for the zebrafish *crash&burn (crb)* mutant [35], we employed a microRNA-mediated knockdown strategy that reduced Ccnb1 to comparable levels (60–80% of controls) present in B-MYB knockdown cells (Figure 2C). In these experiments, no significant differences could be demonstrated in the total number of cells present per colony, BrdU incorporation, the number of cells in G2/M (not shown), or Oct4 expression levels; however, decreased Ccnb1 caused a significant but modest reduction in B-Myb transcripts (p<0.05, n = 4, Figure 2C). Even when Ccnb1 transcripts were reduced by ∼80%, we were unable to detect any evidence of polyploidy, spindle defects or other cell cycle abnormalities (Figure 2D). These results show that, at least in mammalian-derived ESCs, B-MYB leads to cell cycle defects that cannot be fully explained by phosphorylation or the actions of a single known downstream B-MYB target gene that is required for progression through G2/M in somatic cells.

### Alteration of gene expression by B-MYB knockdown

**Figure 3:**
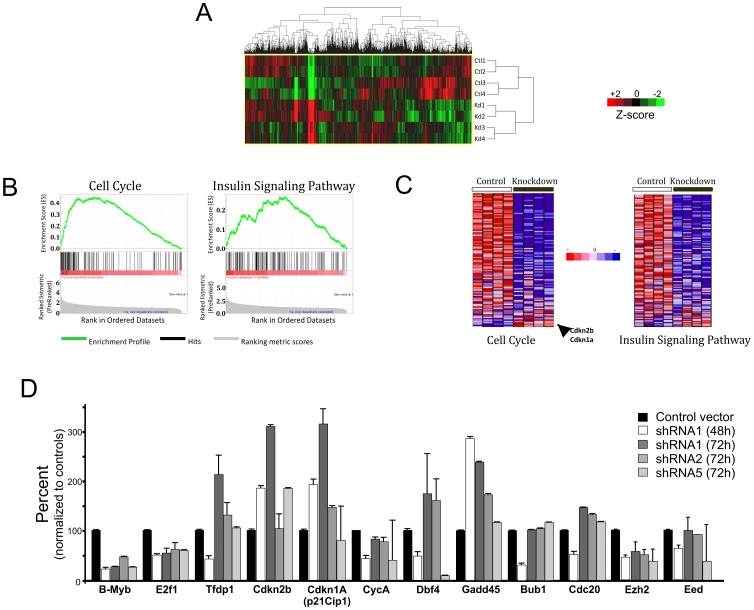
Pathway enrichment analysis. A) Hierarchical clustering of differentially expressed genes (fold-change ≥1.5) in control and B-MYB deficient cells. Red and green colors denote increased and decreased expression, respectively. Each row represents a unique experiment (n = 4, ctl and n = 4, KD) B) KEGG pathway enrichment among the B-MYB knockdown-repressed genes. The enrichment profiles for cell cycle and insulin signaling pathways for genes with reduced expression in B-MYB deficient cells are shown. C) Heatmaps showing gene expression in cell cycle and insulin signaling pathways, indicating that the majority of transcripts are decreased (blue) in abundance in the absence of B-MYB. D) Confirmation of shRNA1 specificity and qPCR analysis of selected cell cycle products and epigenetic regulators. Most of the transcripts analyzed by qPCR showed consistent trends in expression after nucleofection with shRNA1, shRNA2 or shRNA5. Exceptions included Cip1 and Dbf4, both of which had unexpectedly low transcript levels with shRNA5 relative to shRNAs 1 and 2. Almost all of the transcripts with decreased expression 48 hours after nucleofection had modest to significant increases in mRNA expression 24 hours later.

Having established a robust and reproducible model system to knockdown B-MYB in mouse ESCs, genome-wide RNA expression analyses were performed to determine which genes respond to the transient loss of B-MYB. Normalized expression data revealed that B-MYB significantly regulates the expression of 5.5% and up to 18.6% of the ∼25,600 well-annotated RefSeq transcripts present on the Illumina BeadChips. In these experiments, ∼18,000 transcripts were expressed at levels above background in both experimental conditions. A total of 4,768 (18.6%) and 1,407 (5.5%) gene transcripts exhibited ≥1.5 and ≥2.0 fold differences in abundance, respectively between control and B-MYB-deficient ESCs. Among those transcripts with ≥1.5 abundance changes, >95% of the transcripts displayed elevated expression in control ESCs (4,554) relative to those lacking B-MYB. Only 214 mRNAs had increased abundance in the B-MYB deficient cells (Figure 3A, Table S1).

We employed gene set enrichment analysis (GSEA) [36] and Fisher's exact tests to identify biological processes and pathways significantly over-represented in differentially expressed gene sets. A total of 51 enriched KEGG pathways were affected by loss of B-MYB (p<0.05 and FDR q<0.25) (Table S2A). The most significantly enriched biological processes were associated with cell cycle, mitosis and mitotic regulation, chromosome organization and biogenesis, and DNA repair and replication (Table 1). For those genes that showed increased expression following B-MYB knockdown, the biological processes that were most affected are reported in Table S2B, and include cellular defense response, synaptogenesis, cyclic nucleotide mediated signaling and ion transport. Figures 3B and 3C illustrate a GSEA of cell cycle (the most affected pathway) and insulin signaling pathways. Of 103 genes in the cell cycle pathway, 65 were down-regulated (>1.5-fold) following B-MYB knockdown, while 4 were up-regulated, including two inhibitory proteins normally absent in ESCs. Many of the transcripts with reduced abundance encode proteins that regulate G1 to S and G2 to M transitions. GSEA further revealed that TFs E2F, E2F1, E2F1-DP1&2, and E2F4-DP1&2 were significantly enriched (p = 0, FDR q = 0) in gene sets with reduced expression (Table S3), while knockdown of B-MYB specifically reduced the expression (>1.5 fold) of E2F1-E2F5, DP1, and DP2 (Table S1). B-MYB deficiency also altered the core transcriptional program mediated by Oct4, Sox2 and Nanog. These gene products were reduced by 60%, 58% and 46% respectively, while that of B-MYB was reduced by >80% in this experimental set. B-MYB deficiency also significantly reduced expression of key factors that regulate self-renewal and differentiation including those from genes encoding pluripotency-associated TFs (e.g. Tcf3, Tcl1, Esrrb, Zfp281, Zic3, Ctcf, E2f1), Polycomb repressive complex-2 (PRC2) core components (Eed, Ezh2 and Suz12) and co-binding proteins (Jarid2), PcG genes (Phc1 and Rnf2), DNA methyltransferase Dnmt3b, H3K9 demethylase Jmjd2c, and reprogramming factors (Lin28) (see Table S1)(see reviews [37], [38], [39], [40]).

**Table 1:** Biological processes and pathways associated with B-MYB deficiency.

Biological process of gene ontology (GO)	Gene number	P value	FDR q-value
Cell cycle	271	0	0
Cell cycle phase	148	0	0
Cell cycle process	169	0	0
Chromosome organization and biogenesis	104	0	0
DNA repair	112	0	0
DNA replication	85	0	0
M phase	101	0	0
M phase of mitotic cell cycle	75	0	0
Microtubule cytoskeleton organization and biogenesis	30	0	0
Mitosis	73	0	0
Mitotic cell cycle	134	0	0
Protein RNA complex assembly	54	0	0
RNA processing	148	0	0
Translation	147	0	1.45E-04
Protein folding	49	0	1.84E-04
Cellular component assembly	261	0	7.17E-04
Interphase of mitotic cell cycle	52	0	7.63E-04
Golgi vesicle transport	42	0	0.001252277
Intracellular transport	242	0	0.002075577
Regulation of cell cycle	155	0	0.002068629

A total 18,097 genes were ranked by fold-change of gene expression between control and B-MYB knockdown in mouse ESCs based on microarray experiments (see Methods). Ranked genes were analyzed using GSEA based on GO biological processes. The biological processes in the table represents enriched ones with P value of 0 for under-expressed genes by B-MYB knockdown.

To validate the microarray results, we selected 17 transcripts, including B-Myb, with altered expression, and quantified their abundance by qPCR in independent experiments (n = 3). To ensure against non-specific effects associated with the use of shRNA1, RNA was prepared from cells nucleofected with shRNA1, 2 or 5. As shown in Figure 3D, the vast majority of the transcripts showed transient changes in expression within 48 hours (24 hours of puromycin selection) of B-Myb KD. At 72 hours post-nucleofection, most of the transcripts (15 of 17), regardless of the shRNA employed, returned towards control levels, consistent with what was shown in Figure 2 for Plk1. Two exceptions were noted. P21^Cip1^ increased in response to knockdown by shRNA1, but it did not show significant increases in abundance with either shRNA2 or shRNA5 at either 48 or 72 hours. Dbf4 mRNAs transiently decreased in abundance following B-Myb knockdown by shRNA1 and shRNA2, whereas only a sustained decrease was observed with shRNA5. These data, while largely confirmatory of the expression patterns determined by microarray, show that some differences in gene expression may be attributable to non-specific effects associated with shRNA1.

### Intra-pathway transcriptional modulation by B-MYB

**Figure 4:**
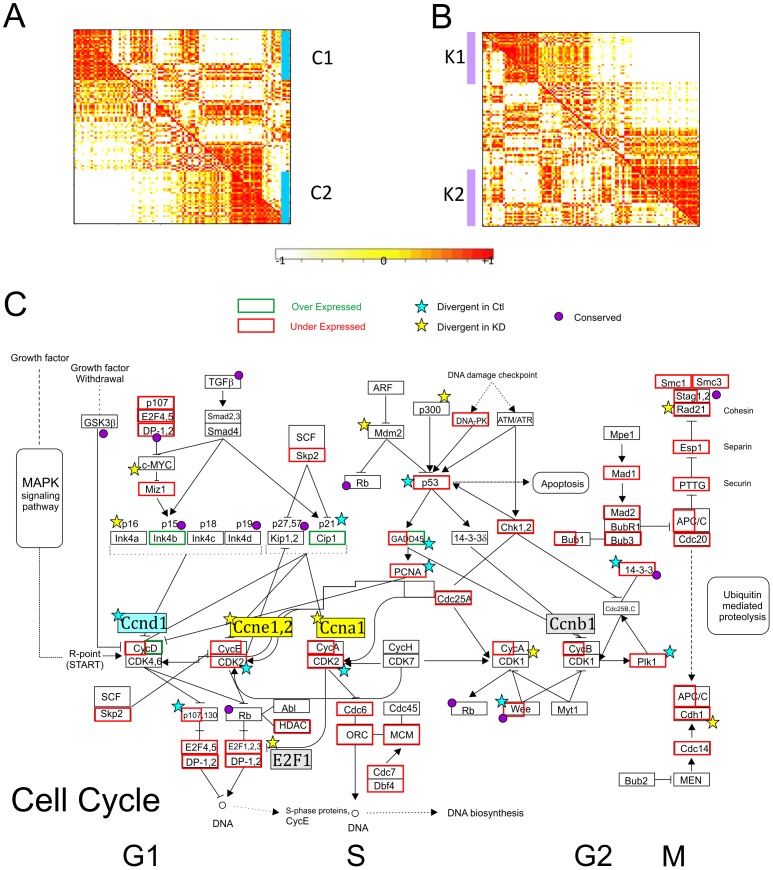
Identification of conserved and divergent co-expression gene clusters in the cell cycle. A) Graphic representation of a correlation matrix of genes based on expression profiles in control (the lower-left triangle) and B-MYB knockdown (the upper-right triangle) ESCs. Genes are listed on the horizontal and vertical axes in the same order. Clustering profiles were determined from the correlation of gene expression where control samples were used as the primary reference. C1 and C2 represent co-expression clusters in controls. The light-to-dense color gradient on the graph indicates low-to-high correlations between gene and their expression. B) Clustering analysis based on the correlation of gene expression where B-MYB deficient (knockdown) samples were employed as the primary reference. K1 and K2 represent co-expression clusters in knockdown conditions. C) Core network illustrating expression and co-expression patterns in a KEGG cell cycle pathway.

Modular behaviors of gene expression are useful at uncovering cellular mechanisms implicated in functional control. By extension, co-expression gene clusters are informative of transcriptional modulation. Figures 4A–4B illustrate the identification of co-expression clusters in the cell cycle pathway, where we identified 2 co-expression clusters in control (C1 and C2) and in B-MYB knockdown (K1 and K2) cells, respectively. We also uncovered 2 conserved and overlapping co-expression clusters, O1 and O2 (Table 2, Table S4A). Within the cell cycle network, a number of genes showed conserved co-expression patterns, which were unchanged after B-MYB knock-down. These conserved co-expression modules included inhibitory genes like cyclin-dependent kinase inhibitors (Cdkn2b, Cdkn2d, Cdkn1b and Cdkn1c) and cyclin D inhibitor Gsk3β in G1 and S, and cyclin inhibitors (Gadd45A-B, 14-3-3s) and Wee1 in G2 and M phases (Table S4A), indicating that B-MYB does not directly impact the *co-regulation* of these core inhibitory genes in ESCs. In contrast, numerous cell cycle core members showed divergent co-expression patterns, where genes showed unique relationships depending on the experimental condition. For example, under conditions where B-MYB is normally expressed (i.e., controls), transcriptional co-expression was observed among G1/S associated cyclin D1, kinase Cdk2 and their targets Tfdp2 and Rbl1 (p107); Trp53 and its target gene Cdkn1A (p21^Cip1^); and the G2/M transition protein Gadd45G that inactivates the cdc2-cyclin B complex. This co-expression pattern was not, however, maintained in B-MYB deficient cells (Figure 4C). Similarly, Ccne1 and Ccne2, E2f1, Skp1a, and Ccna1 during G1/S transitions, as well as the p53 cofactor CBP/p300 were co-regulated in normal cells, but uncoupled in B-MYB deficient cells (Table S4A). This divergent co-expression pattern indicates that B-MYB significantly modulates the transcriptional- and co-regulation among core cell cycle elements.

**Table 2:** Identification of co-expression gene clusters in ES cell critical pathways.

Pathway	No of genes in the pathway	Co-expression cluster	No of genes in the cluster	Average r (correlation coefficient)	No of differentially expressed genes in the cluster (under-/over-expressed by B-MYB knockdown)
Cell cycle	116	C1	30	0.735	18/0
		C2	32	0.776	16/3
		K1	30	0.747	19/0
		K2	36	0.767	16/1
		O1	21	0.718(ctl)/0.738(kd)	11/0
		O2	19	0.838(ctl)/0.777(kd)	8/1
Apoptosis	78	C1	28	0.719	6/0
		C2	16	0.716	2/0
		K1	28	0.669	4/0
		O1	15	0.717(ctl)/0.816(kd)	2/0

C1–2 and K1–2 represent co-expression clusters identified in the ES cells of control and B-MYB knockdown, respectively. O1–2 represents conserved co-expression clusters cross the control and B-MYB knockdown. Ctl: ES cell control, kd: ES cells with B-MYB knockdown.

Apoptosis genes also showed considerable change in transcriptional co-expression in response to B-MYB deficiency, consistent with our previous report of apoptosis [27] (Table S4B). Among 78 apoptosis genes examined, 20 were significantly down-regulated while only 4 were up-regulated in B-MYB deficient cells. Both apoptotic (Apaf1, Atm and Casp9) and anti-apoptotic (Birc2, Rela and Irak) genes showed conserved co-expression. The ligands Tnfα and Fasl and their receptor Tnfsf1a however showed divergent co-expression, as did Trp53, and pro-apoptotic (Bid, Casp3, 6–8) and anti-apoptotic (Bcl-xl and Bcl-2) transcripts (Table S4B). Some important apoptotic genes (i.e. *Trp53, Casp3&6*, and *Bid*) were significantly down-regulated by the loss of B-MYB and were co-expressed in a divergent pattern. Moreover, p53 target genes *Bbc3* and *Perp* showed co-expression in B-MYB knockdown cells but not in control cells. B-MYB deficiency therefore has a broad impact on co-expression patterns of pro-apoptosis and anti-apoptosis genes, and is actively involved in the transcriptional modulation of apoptotic genes in ESCs.

### Transformation of the global transcriptional network by B-MYB knockdown

We constructed global co-expression networks based on transcriptional correlations (i.e., expression patterns) from all genes expressed in normal (16,058 genes) and B-MYB deficient ESCs (16,143 genes), respectively. The resulting networks, composed of nodes (i.e, genes) and links (i.e, co-expression between genes), showed scale-free topology (Figure S1), indicating that each was dominated by a small set of highly-connected genes (hubs) that link less connected genes to the system (Table S5). Strikingly, most of the hub genes in the co-expression network of B-MYB deficient cells differed from the hub genes of controls (Table S5A–D). As an example, B-Myb functions as a hub gene in control cells, but in B-MYB deficient cells, it no longer functions as a hub gene. Among the 300 most prominent hub genes in each network, only 51 genes were in common, while among the top 100 hub genes, only 1 gene was present in both networks. This lack of hub similarity indicates that B-MYB is critical to the maintenance of essential biological functions in ESCs.

**Figure 5:**
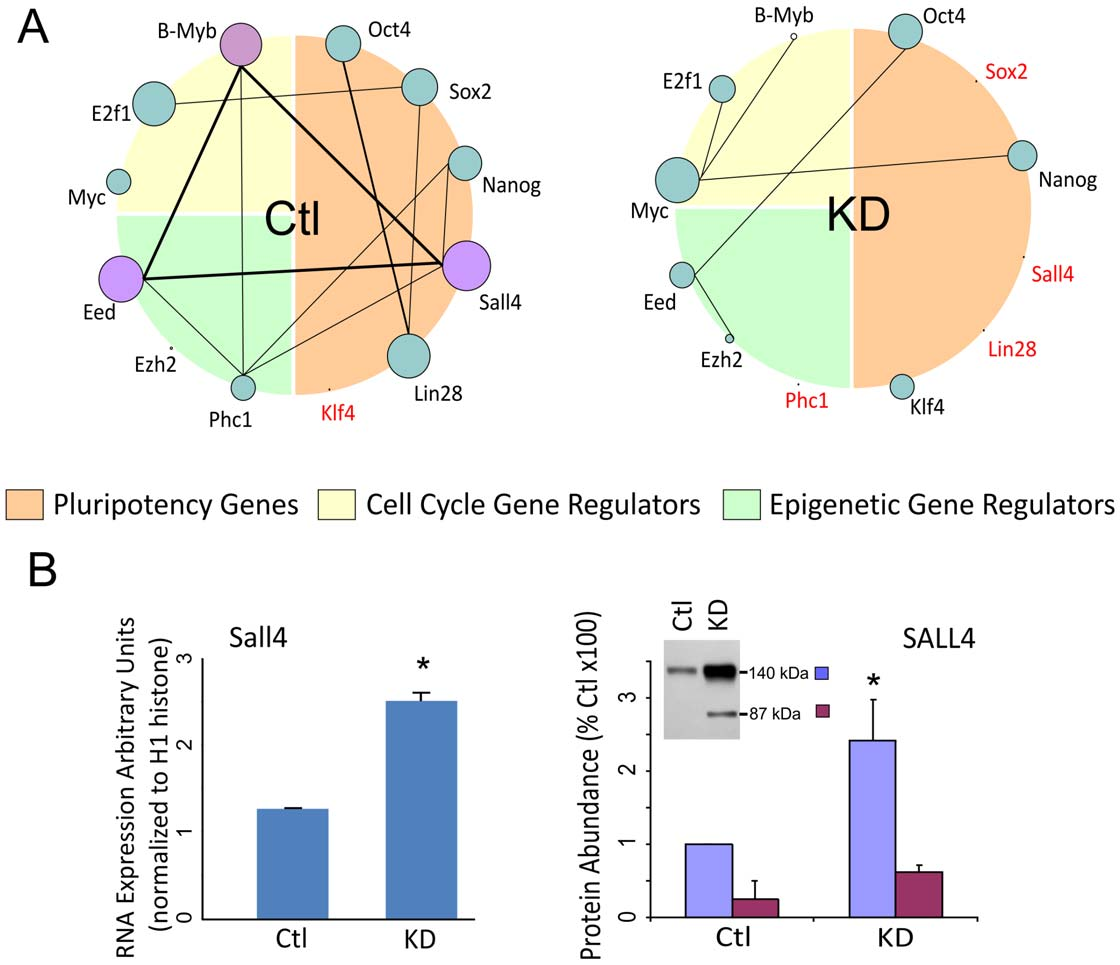
Global network analysis showing the distribution of connections per node in the co-expression networks. A) Interactome showing hub genes and connectivities among hubs of selected pluripotency, cell cycle and epigenetic regulator-associated genes in control (Ctl) and B-Myb knockdown (KD) cells. The size of each circle is directly proportional to the number of links (connectivity) for the indicated hub gene, and lines between genes are indicative of the number of overlapping links. As shown in this figure, B-Myb, Eed and Sall4 are major hub genes in control cells that have overlapping links with over 160 genes. Following knockdown of B-MYB, all of major hub genes either become minor hubs or lose all connectivity (shown in red). B) Based on the analysis, Sall4, a transcription factor found to be central to the control ESC network, loses all connectivity in B-MYB deficient ESCs (4A); but in response to the loss of B-MYB, Sall4 transcript and protein abundance increase significantly, suggesting that the up-regulation of this gene is a compensatory response to the loss of B-MYB.

Many of the network genes, including hub genes, showed considerable changes in connectivity (i.e. the number of links to each gene) in response to B-MYB knock-down (Table S5C,D). Altered connectivity was particularly important to genes encoding TFs (Sox2, Lin28, Klf4), epigenetic regulators (Phc1, Eed, Ezh2), and signaling molecules (Tcf7, E2f1, Smad3) involved in ESC developmental processes. For example, B-Myb, Eed and Sall4 were among the top 300 hub genes in control ESCs, but following knockdown, their connectivity, and consequently functional importance, were significantly reduced (Figure 5A). In contrast, the network connectivity of the cell cycle regulatory gene c-Myc and pluripotency factor Klf4 significantly increased; however, none of these factors remained among the top 300 hub genes of B-MYB deficient cells. The single TF hub gene with the largest change in connectivity was Sall4, even though its expression increased following B-Myb knockdown. It was connected to a total of 959 genes in normal cells, while no links remained in B-MYB deficient cells (Table 3). Similarly, Sox2 and Lin28 showed a dramatic reduction in their connectivity; but unlike Sall4 (Figure 5B), these gene products were reduced in response to B-MYB knockdown. Other examples of hub genes present in control but not B-MYB deficient cells included hedgehog pathway gene Smo, apoptotic Igfbp3, anti-apoptotic Bcl-xl, Wnt and TGFβ pathway genes Ppp2r1a and Ppp2cb, Wnt genes Chd8 and Nfact4, and notch receptor Notch3. While B-MYB deficiency decreased network connectivity on those genes, it increased the network connectivity of pluripotency regulators c-Myc, Klf4, Tcf3, Ezh2, Jmjd1a, Rnf2, and Dnmt3b (Table 3). B-MYB deficiency did not, however, significantly alter network connectivity of pluripotency factors Oct4 and Nanog, even though the expression level of both was significantly reduced.

**Table 3:** Connectivity and fold-changes of key regulatory genes in the co-expression networks of mouse ESCs.

Gene symbol	Regulator	Fold change	Connectivity in the control	Connectivity in the knockdown
Sall4	TF	1.165	959	0
Mybl2	TF	4.815	867	36
Tcf7	TF	1.881	773	0
Lin28	Post-transcriptional	2.955	763	0
Sox2	TF	2.409	521	0
E2f1	TF	1.888	685	211
Eed	Epigenetic	1.633	916	471
Smad3	TF	1.056	468	87
Suz12	Epigenetic	1.616	430	213
Phc1	Epigenetic	1.732	193	0
Zfx	TF	1.464	477	294
Nanog	TF	1.778	464	343
Jmjd2c	Epigenetic	1.667	305	223
Oct4	TF	2.501	682	665
Jarid2	Epigenetic	1.645	0	7
Trp53	TF	1.766	0	16
Dnmt3a	Epigenetic	1.053	333	352
Stat3	TF	1.224	0	47
Smad1	TF	1.239	14	86
Ctcf	TF	1.681	229	326
Tcl1	TF	3.490	148	261
Zfp281	TF	1.676	38	275
Esrrb	TF	2.172	139	431
Klf4	TF	1.331	0	293
Jmjd1a	Epigenetic	1.385	631	925
Tcf3	TF	1.885	497	806
Rnf2	Epigenetic	1.786	0	392
Ezh2	Epigenetic	2.508	4	404
Dnmt3b	Epigenetic	2.393	204	635
c-Myc	TF	1.095	186	742

Fold changes are calculated as the ratio of expression between control and B-MYB deficient ESCs. Connectivity is the number of connected genes for each gene on the network. Green highlights factors with reduced connectivity, while red indicates factors with enhanced connectivity among genes.

Finally and when assessing changes in connectivity, it is important to realize that connectivity is not necessarily correlated with the magnitude of a fold change determined by gene expression analyses. Our microarray data specifically demonstrated that B-MYB knockdown results in differential gene expression and fold changes in RNA, i.e., how B-MYB influences the expression of *individual genes*. In contrast, connectivity shows *gene-gene relationships* in terms of co-expression patterns under two conditions. A high value of connectivity indicates how many genes may be linked with a gene based on a similar expression pattern. Table 3 clearly shows that several important ESC regulators (e.g. Sox2, Lin28, Tcl1, Esrrb, Ezh2, Dnmt3b) show consistent changes in fold change and in connectivity, while other genes (e.g., Oct4 and Nanog) show alternation in one but not the other.

### B-MYB target genes by ChIP-chip identification

**Figure 6:**
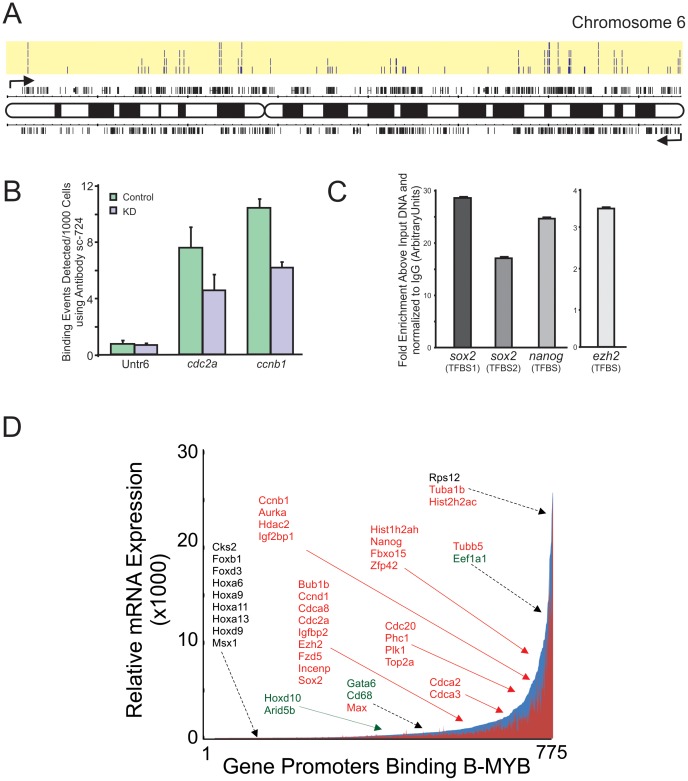
Identification of B-MYB binding sites on gene promoters and corresponding gene expression profiles. A) ChIP-chip analysis showing the locations and number of B-MYB binding sites (highlighted in yellow shading, n = 4 biological replicas) and corresponding genes on Chromosome 6 (for details, see Table S6). Binding was determined from promoter regions corresponding to 8 kb upstream and 2 kb downstream of the 25,500 transcription start sites of the whole mouse genome. B) To determine antibody specificity, ChIP assays of known B-MYB target genes were performed in ESCs after B-MYB knockdown. Relative to controls, the number of binding events on known target genes *cdc2a* and *ccnb1* was significantly reduced in B-MYB deficient cells (KD), indicating that the antibody had good specificity for B-MYB. Untr6 – Untranscribed control DNA sequence. See Methods for experimental details. C) Independent ChIP assays using IgG precipitated chromatin as a control confirming binding of B-MYB to the promoter regions identified by ChIP-chip for *sox2 (two sites), nanog* and *ezh2*. D) Plot of B-MYB target genes (Table S6) with corresponding microarray expression data taken from Table S1. This plot indicates that B-MYB target genes have expression levels ranging from just detectable to elevated.

To identify B-MYB target genes that could account for the cell cycle abnormalities and changes in gene transcription described above, chromatin immunoprecipitation (ChIP-chip) experiments were performed. An example of B-MYB binding sites along Chromosome 6 is illustrated in Figure 6A. The data revealed that up to 8.2% of known promoters in the mouse genome bound B-MYB. Four independent analyses of R1 ESCs identified a total of 2250 promoter regions that bound B-MYB in at least one sample, 1020 promoters in 2 samples, 482 promoters in 3 samples, and 184 promoters in all 4 samples (Table S6). A number of individual gene promoters bound B-MYB in more than one discrete site (i.e., Sox2); whereas the majority bound B-MYB at only one location or on closely overlapping “tiles”. In B-MYB deficient cells, the number of detectable promoter sites that bound B-MYB above threshold was reduced by 77.1% relative to controls (CHiP-chip), and a 60% reduction was observed in the total number binding events detected for two control genes Cdca2 and Ccnb1 by CHiP (Figure 6B). Antibody immunoprecipitation of B-MYB chromatin binding was therefore highly specific and significantly reduced in B-MYB deficient cells. The results further revealed that pluripotency genes *sox2* and *nanog* bound B-MYB, but no binding to the promoter of *pou5f1* (Oct4) was observed. Independent ChIP assays, where data were normalized using an independent IgG based technique, confirmed binding of B-MYB to the promoter regions identified by ChIP-chip for *sox2 (two sites), nanog*, *ezh2* and *lamb2*, among others (Figure 6C). Importantly, most B-MYB target genes were primarily associated with biological processes corresponding to those observed from the transcriptome-based study described above, strongly suggesting that the changes in RNA abundance were directly attributable to altered transcriptional activity mediated by B-MYB (Table 4 and Table S7).

**Table 4:** Significantly enriched biological processes and pathways among the informative B-MYB target genes identified from CHiP-chip and microarrays.

Biological process of gene ontology (GO)	Gene number	*P* value	FDR
Mitosis	43	7.35E-21	1.28E-17
M phase of mitotic cell cycle	43	1.70E-20	2.97E-17
M phase	51	4.12E-20	7.20E-17
mitotic cell cycle	45	3.53E-18	6.16E-15
cell cycle phase	52	5.09E-18	8.89E-15
cell cycle process	56	3.00E-17	5.24E-14
DNA packaging	29	4.60E-17	8.04E-14
cell cycle	69	1.13E-15	1.94E-12
protein-DNA complex assembly	22	4.65E-13	8.12E-10
chromatin organization	39	3.12E-10	5.45E-07
regulation of transcription	137	5.50E-09	9.61E-06
embryonic morphogenesis	35	1.03E-06	0.0018054
cell fate commitment	18	4.58E-05	0.0800091
cell differentiation	88	1.99E-04	0.3476293

B-MYB target genes were identified by comparisons of the microarray and ChIP-chip datasets. Significance was determined using Fisher's exact tests (see materials and Methods). A total 775 target genes expressed in the microarray experiments was analyzed by DAVID based on GO biological processes (*P*<0.05, FDR<0.5).

Computational analysis of the promoter sequences that bind B-MYB identified 50 consensus cis-elements, whose presence differs significantly from predicted values (P≤0.01) (Table S8). The most over-represented *cis*-elements (Z-score = 21.54) corresponded to the cellular and viral myb-like transcriptional regulators (*mybl* binding sites), which was present in a total of 1540 (61%) input sequences. *Cis*-elements to E2F-myc activator/cell cycle regulator (Z score = 6.52), SOX/SRY-sex/testis determining and related HMG box factors (Z score = 6.93) and paralog hox gene (Nanog) binding factors (Z-score = 7.85) were significantly over-represented, as were cis-elements to homeodomain- and homeodomain leucine zipper TFs (Table S8). Cis-elements to E-box binding factors like C-MYC (Z score = −6.29) were significantly under-represented in promoters that bind B-MYB. No single binding site was conserved among all identified gene promoters, and among the B-MYB bound promoters, almost half did not contain an IUPAC consensus *mybl* binding site; however, a potentially novel IUPAC consensus sequence of NAAAANAAAN was present in 76% of the promoters.

### Integrative analyses of ChIP, microarray and methylation data

Integrative analysis of gene expression, TF binding and epigenetic data is a powerful approach for determining functional relationships in gene networks. We, therefore, identified an informative gene set based on the overlap between B-MYB binding targets and whole-genome expression data. Among 1020 B-MYB target genes identified by ChIP-chip and present on microarrays, 775 (76%) were expressed in ESCs at levels equal to or above threshold. 361 were differentially expressed by at least 1.5-fold, and 96% of differentially expressed genes showed reduced expression following B-MYB knockdown (Table S9). The most prominent biological processes associated with this informative set centered on mitosis and cell cycle regulation, but morphogenesis, cell fate commitment, and differentiation were also significantly enriched (Table 4). Conclusively, cell cycle genes were significantly over-represented (P = 1.71×10^−4^, FDR = 0.20). Notably, 16 of 361 differentially expressed B-MYB target genes belonged to the cell cycle pathway and included G1 proteins Ccnd1, Cdk6 and Trp53, and G2/M proteins Ccnb1, Cdc25c, Wee1, Plk1, and Bub1b. Apoptosis pathway genes (Trp53, Birc2, Casp3), pluripotency factor genes (Sox2 and Nanog), PcG gene Phc1, core PRC2 component Ezh2 and its co-binding protein Jarid2, and H3K9 demethylase Jmjd2c were also present in this informative and differentially expressed target gene set (Table S9). Transcript abundance was highly variable among the informative 775 B-MYB target genes (Figure 6D).

**Figure 7:**
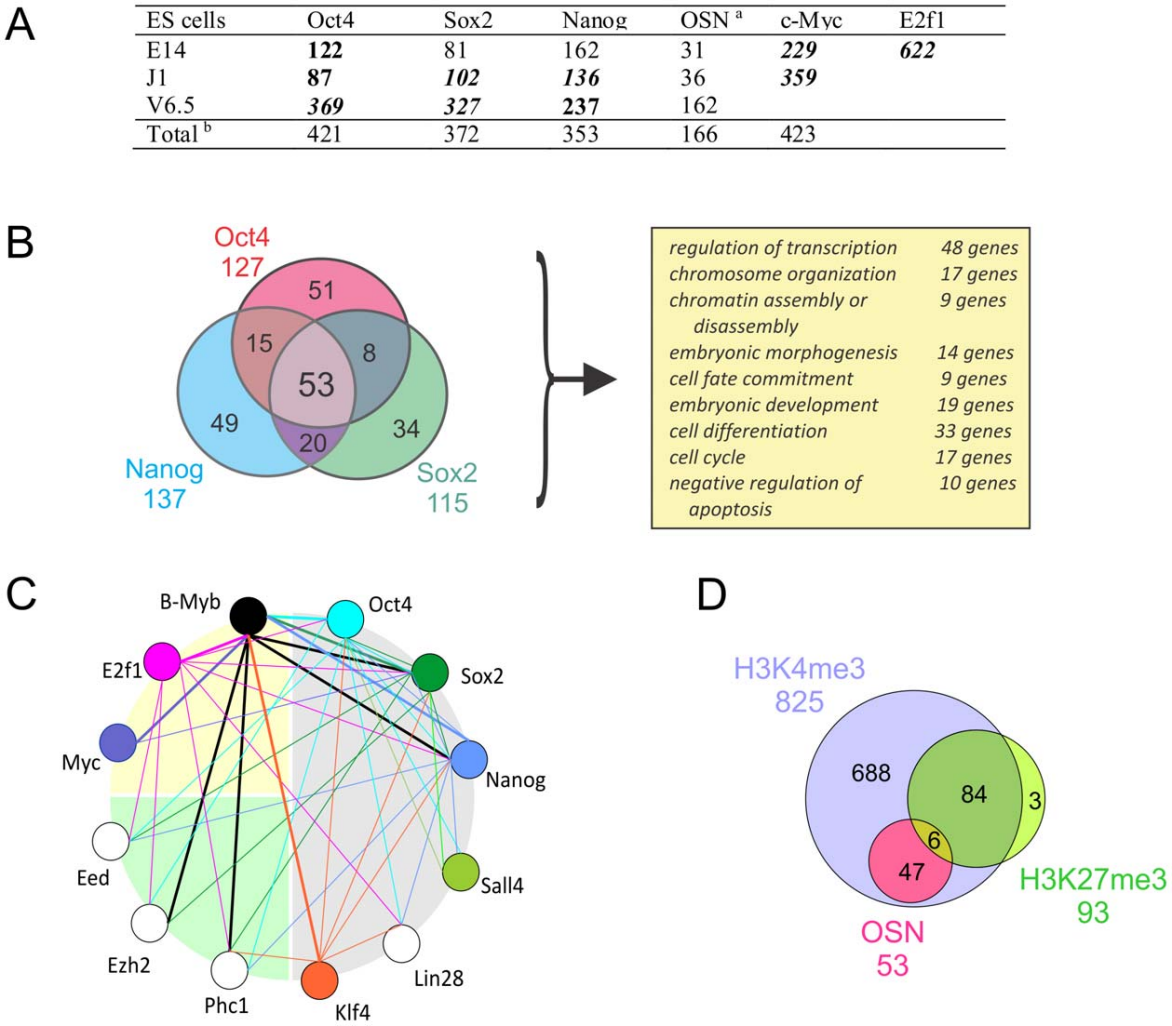
B-MYB target genes and possible interactions with pluripotency factors and regulation by histone methylation. A) Overlap of target genes between B-MYB and other selected TFs (see materials and Methods). Numbers in bold and italic refer to significant overlaps between two gene groups with p<10^−10^, in bold refers to p<10^−3^, respectively. B) Overlap of binding sites for Oct4, Sox2, Nanog (OSN) and B-MYB, and the corresponding biological process (GO terms) that were significantly elevated among the overlapping genes. C) Transcription factor binding interactome of selected genes associated with pluripotency, cell cycle and epigenetic regulation. Each gene is indicated by a circle, and lines connecting genes indicate TF binding. TF binding is directional, as the color of the line indicates which factor binds to the connected gene promoter. The data indicate a high level of crosstalk among pluripotency, cell cycle and epigenetic regulators with B-Myb. Data were taken from Table S1. D) Overlap among B-MYB target genes with OSN (common targets of Oct4, Sox2 and Nanog identified in at least two ES cells) and the state of histone methylation (H3K4me3, H3K27me3). All target genes used in this analysis were differentially repressed by the B-MYB knockdown based on the microarray experiment.

To better define how B-MYB target genes are regulated in ESCs, we identified genes co-targeted by B-MYB, OCT4, SOX2 and Nanog (OSN, Figure 7A and Table S1). We determined that 35–41% of the B-MYB target genes bound OSN, 166 of which (16%) bound multiple pluripotency TFs (p<0.05), and 53 target genes were putatively regulated by all four TFs (Figure 7B), (e.gs., *zfp42*, *sox2*, *nanog*, *trp53*, *phc1* and *jarid2* (Table S1)). Genes, like *ezh2*, bound B-MYB, OCT4, and SOX2, but not NANOG, while *fbxo8* and *tuba1a* only bound B-MYB. Co-targeted genes were significantly associated with stem cell differentiation, embryonic development, regulation of transcription, and epigenetic control; however, cell cycle-related proteins, while present, were under-represented among the informative co-targeted gene set (Figure 7A). Consistently, many of the genes that bound only B-MYB included cell cycle pathway specific genes (Table S10), as well as cell-cycle associated proteins involving mitosis, centrosome and spindle formation, DNA and RNA polymerases, and ubiquitin-specific peptidases (Figure 7B, Table S1). B-MYB, therefore, co-targets a set of genes bound by multiple pluripotency TFs, and it has unique targets that are not regulated by OSN.

When compared with 15 other TFs implicated in self-renewal and pluripotency, the largest intersection of target genes was with E2F1 (61%) (Tables S1 and S10). This TF bound to all 16 B-MYB target genes associated with cell cycle regulation, including those that did not bind OSN. We have not, however, been able to detect any direct physical interaction between B-MYB and E2F1 in ESCs by immunoprecipitation, Western or mass spectrometry (not shown). Similarly, c-MYC bound to 22.5–35.2% of the B-MYB targeted genes, including *bub1b, ccnb1*, and *plk1* (Table S1). Altogether, c-MYC bound to 42 of 116 cell cycle genes; whereas, E2F1 bound to 93 gene promoters, including *trp53, ccnb1, wee1, cdc20 and plk1*. These latter data indicate probable transcriptional interactions between B-MYB and E2F1 in regulating cell cycle processes in ESCs, while the potential transcriptional interactions with MYC proteins are somewhat less likely. Perhaps more importantly, the genes encoding the pluripotency factors, cell cycle regulators and epigenetic components associated with the global networks described earlier all show significant and overlapping promoter binding patterns with each other. B-MYB, for example, binds to *sox2* and *nanog*, while OSN, E2F1, c-MYC and KLF4 all bind to the *mybl2* gene promoter (Figure 7C).

When compared with known histone methylation sites and ChIP-seq data from mouse ESCs V 6.5, we found that >80% of the 1020 B-MYB target genes were H3K4 trimethylated, and that 95% (734) of the 775 informative B-MYB target genes contained H3K4me3 markers (Figure 7D, Table S9). Conversely, only 93 (9%) putative B-MYB target genes contained H3K27me3 markers, and only 9 of these had reduced expression following B-MYB knockdown. Included among the bivalent genes with H3K27me3 marks were known regulators of differentiation and specification like Gata6, Hoxa6, Hoxa9, and Hoxd11. Importantly, 88.7% of the OSN and B-MYB co-targeted genes were marked by active histone methylation (H3K4me3) (Figure 7D), and as described earlier, most of these had reduced expression in B-MYB knockdown cells. B-MYB target genes that did not show significant decreases in expression in the absence of B-MYB (Figure 6D, Table S9) had a preponderance of H3K27me3 markers (P<0.001).

## Discussion

The quintessential stem cell trait of self-renewal requires coordination of cell cycle progression with fate choices [16]. In PSCs, differentiation is believed to be actively suppressed by coincident activating and silencing histone modifications (i.e., a bivalent gene), and by promoter binding of pluripotency-promoting factors OSN. Here and consistent with our previous report [27], we demonstrate that B-MYB is a critical regulator of the ESC cell cycle, as deficiencies in this TF lead to functional defects in S, G2 and M phases and to transcriptional modulation of genes involved in the control of all cell cycle phases in ESCs. The results also show that B-MYB primarily up-regulates gene activity and specifically regulates, either directly or indirectly, genes encoding pluripotency factors, chromatin and histone modifiers (including PcG proteins), signaling molecules, and TFs involved in fate choices. The altered expression of these targeted genes has widespread consequences that ultimately affect at least 5.5% of the entire ESC transcriptome. These results not only link B-MYB to cell cycle progression and fate decision (i.e, self-renewal), they demonstrate that B-MYB in conjunction with other pluripotency factors and DNA modifying enzymes are integral to the network that maintains ESC homeostasis and the ESC phenotype. More specifically, it is the coordinated interactions among pluripotency TFs, histone methylation and B-MYB that maintain the expression of most B-MYB target genes; however, B-MYB, E2F1 and perhaps c-MYC, play a preeminent role in the control of DNA replication and cell cycle progression.

### Cell cycle control and self-renewal

In this study, we have begun to unravel the molecular basis for ESC cell cycle control of self-renewal mediated by B-MYB. By genome-wide transcriptome profiling, we identified a broad spectrum of cell cycle genes repressed in B-MYB deficient cells. Through gene co-expression profiling, we demonstrated that B-MYB transcriptionally modulates key members of G1 (Ccnd1, Cdk2), S (Ccne1&2, Ccna1), and G2/M (Cdh1, Cdc20 APC/C) phases in ES cells, and that co-expression patterns become uncoupled in B-MYB deficient cells. We also determined that functional defects observed following loss of B-MYB could be directly associated with altered expression of key cell cycle components. Centrosome and spindle defects, for example, may be due to B-MYB targets associated with CENP-A NAC/CAD kinetochore complex dysfunction or regulatory protein abnormalities involved in mitosis control (Table S1) [41], [42], [43].

This study also unveiled a strong association between the decreased expression of genes regulated by B-MYB with E2F1 and to a lesser extent c-MYC. A significant reduction in expression of genes encoding E2F1-5 and DP1,2 (Table S1), as well as a significant loss in E2F1 network connectivity were observed in B-MYB deficient cells. B-MYB target gene promoters contained significantly elevated numbers of E2f binding sites, and a majority of these genes bound E2F1 TFs [44], including most cell cycle genes (93 genes; 80% of the total) and all 16 cell cycle pathway genes targeted by B-MYB (Tables S1 and S10). E2F1, however, binds to a much broader gene set than B-MYB, and only 68% of the genes repressed in B-MYB deficient cells bind E2F1 (mouse ES cell line R14 ChIP-seq comparisons, data not shown). These findings are consistent with overlapping and cooperative interactions between B-MYB and E2F1 [45], [46]. Significant overlap also exists between gene promoters that bind c-MYC and B-MYB, including 13 of 16 B-MYB target genes present in the cell cycle pathway. B-MYB neither binds to the *c-myc* promoter, nor significantly alters the abundance of c-Myc in ESCs lacking B-MYB. It does, however, bind to and modulate the expression of its co-factor MAX, and c-MYC binds to the *mybl2* gene promoter. Moreover, the connectivity of c-MYC and its rank among hub genes in the co-expression network are significantly elevated in B-MYB deficient cells. This increase in connectivity is insufficient to completely maintain self-renewal processes, as loss of B-MYB leads to profound cell cycle defects and growth suppression. Moreover, B-MYB promotes an up-regulation of p21^Cip1^ and p15^INK4b^ and a down-regulation of p19^INK4d^; whereas, c-MYC is thought to repress the activation cell cycle inhibitors p21^Cip1^ and p27^Kip1^ [18], [25]. Thus, c-MYC like E2F1 serves as an indirect co-regulator of B-MYB to regulate cell cycle progression and self-renewal processes, but its mechanism of action appears to be distinct from B-MYB and insufficient to account for B-MYBs influence on self-renewal.

### The interplay of B-MYB with transcription regulators of ESC fate

Collectively, our data indicate that B-MYB is implicated in the control of ESC fate decisions (i.e., differentiate or remain pluripotent) through combinatorial interactions with pluripotency-promoting TFs and co-regulators. B-MYB binds to *sox2* and *nanog* promoter regions, and knockdown of B-MYB results in a transient decrease in OSN. Moreover, the *mybl2* gene promoter binds all three of these pluripotency TFs (Table S1) [44], [47], [48], which together with at least 15 other co-regulators drive the core pluripotency network. This newly identified gene circuit has broad implications for ESC biology, particularly since it should be auto-regulatory and bi-stable. Most members of this gene circuit are down-regulated in the absence of B-MYB. For example, network connections with iPSC reprogramming factors Sox2 and Lin28, were completely lost in B-MYB deficient cells, while that of Klf4 was significantly increased.

The results from this study also distinguish the role of B-MYB from that of pluripotency-promoting TFs. First, Sall4 is the *dominant* hub gene in the global co-expression networks of control ESCs (Figure 5A). SALL4 also showed enhanced abundance in B-MYB deficient cells, but after B-MYB knockdown, all links of Sall4 to the network were completely lost. Importantly, the *sall4* promoter is co-occupied by OSN [49], [50], but it is not bound by B-MYB. Since this TF plays a critical role in maintaining mouse ESC pluripotency through transcriptional activation of Oct4 [49], [51], [52], [53] and as a co-regulator in the core TF-driven regulatory network [54], Sall4 must serve as an essential, but indirect bridge linking pluripotency factors to co-expressed genes regulated by B-MYB. Second, it is also noteworthy that *mybl2* and *pou4f1* gene knockouts lead to similar phenotypes in mice, but only loss of Oct4 in ESCs results in a comparable phenotype in vitro and in vivo. Both *mybl2* and *pou4f1* knockouts lead to early embryonic lethality and loss of ICM. Embryos require B-MYB for formation of the ICM; however, it is not required for trophectoderm formation and expansion [26]. Mouse embryos that are Oct4-deficient also fail to form ICM, lose pluripotency and differentiate into trophectoderm. The knockdown phenotype of B-MYB in vitro is however unique to that of Oct4, Sox2 and Nanog. Loss of Oct4 in ESCs favors production of trophectoderm characterized molecularly by the expression of Cdx2, while its overexpression leads to endoderm formation [55], [56]. B-Myb over-expression has very little effect on mouse ESCs, perhaps due to tight regulatory mechanisms that prevent elevated levels of B-MYB proteins in stable clones [57]. By comparison, loss of Nanog promotes primitive endoderm formation (Gata6 expression), while loss of Sox2 produces a variety of changes in vitro and fail to develop an epiblast in vivo [1]. In contrast, knockdown of B-MYB in ESCs results in either pluripotent and aneuploid cells or a delayed and transient increase in a variety of differentiation markers (Cdx2, Hand1 and Sox17) that correlates with increased apoptosis [27]. Third, Oct4, Sox2 and Nanog expression are almost completely repressed within a few days of in vitro ESC differentiation; whereas, B-Myb expression becomes tightly coupled to cell cycle regulation. Moreover, the functional role of B-MYB in pluripotent cells is unique from its role in differentiated cells. In embryonic carcinoma cells, B-MYB is constitutively active, but upon differentiation, it requires the DREAM complex for transcriptional control [58]. The role of B-MYB in maintaining pluripotency is therefore secondary to its effects on cell cycle, and it is likely that B-MYB exerts its effects on pluripotency indirectly through the regulation of other critical pluripotency factors like SALL4.

### The interplay of B-MYB with epigenetic regulators of ESC fate

A unique and potentially critical finding from this study is the role of B-MYB in controlling epigenetic regulators of chromatin. Approximately half of the B-MYB binding sequences identified in this study are characterized by the presence of RNA Pol II binding and H3K4me3, and excluding those that contain H3K27me3 marks (i.e., poised DNA), most of these sequences are transcriptionally active. Moreover, 95% of actively transcribed genes are repressed by knockdown of B-Myb in ESCs. Because H3K4me3 has been proposed as a chromatin mark of transcription initiation [40], [59], [60], B-MYB is either involved in activated expression of target genes or alternatively this mark permits binding of B-MYB to enhance transcriptional activity. These data, along with published studies for OSN, c-Myc and E2F1, suggest that these TFs act coordinately to mediate gene activity that is conducive to pluripotency, differentiation repression, cell cycle progression and consequently to the stem cell trait of self-renewal.

The finding that B-MYB regulates the RNA expression of critical epigenetic regulators (Suv420h2, Phc1, Eed, Ezh2, Jarid2) further defines a B-MYB regulatory circuit that influences ESC homeostasis and differentiation. Each factor is involved in histone methylation events that control fate decisions. Phc1 is a component of polycomb complex PRC1 that is involved in silencing of developmental genes [59], [61], [62], while Jarid2 binds DNA and mediates the recruitment of the PRC2 complex to target genes, but its inhibition of methylation plays a key role in differentiation and development [63], [64]. Moreover, Phc1 and Jarid2 act as hub genes in the global co-expression network conserved between human and mouse ES cells [65]. EED and EZH2 are major components of the PRC2 complex, which methylates Lys9 and Lys27 of histone H3, to repress genes like HOXA7, HOXB6 and HOXC8 [66], [67], [68]. EZH2, in particular, regulates self-renewal processes and proliferation in stem cells from other systems [69], and its connectivity as well as that of Jmjd1a and Dnmt3b are significantly increased in B-MYB deficient cells. Since histone modification levels can be used to predict gene expression levels [37] and both histone and DNA methylations are known to be critical for correct differentiation of ESCs into specific lineages, these findings highlight B-MYB regulatory interactions among pluripotency TFs and histone modifiers in promoting gene expression and controlling ESC fate decisions critical to self-renewal.

### Knock-down model system

The functional and bioinformatics data presented in this paper extend our previously published data that showed punctate and altered BrdU incorporation during S phase, an accumulation of cells in G2/M with spindle defects and polyploidy, and changes in Oct4 abundance. Lorvellec et al. subsequently showed that B-Myb ablation leads to stalling of replication forks and activation of replication factories in part due to c-MYC and FOXM1 regulatory mechanisms. These findings are consistent with the defects in G1 and S phase-associated cell cycle gene expression that we report here, and our co-expression and promoter analysis results implicate c-MYC in B-MYB-mediated gene control. In contrast with our original study and validated RNA data on Oct4 expression, Lorvellec et al. did not observe any influence on *oct4* gene regulation by B-MYB. In this model system, however, we only observed transient decreases in Oct4 and Sox2 following knockdown of B-Myb, which we originally attributed to the transient but rapid decrease in B-MYB caused by shRNAs. While a potential transient loss of Oct4 was not tested by Lorvellec et al., the results from their knockout system suggest model differences that should be considered when interpreting the data presented here. First, some of the transient effects on pluripotency gene expression demonstrated here may be secondary to changes in the proliferation rate and total cell numbers as opposed to the direct regulation by B-MYB. Second, some differences between the knockdown and knockout models may be real but model specific. For example, the acute loss of Rb in mouse embryonic fibroblasts or cardiomyocytes has been shown to induce cell cycle re-entry and cell proliferation; however, germline loss or knockout of Rb does not [70], [71]. These differences can be explained, at least partially, by a functional compensation with other pocket proteins (p107) present in animal models that does not occur in the cells [70]; however, neither A-MYB nor C-MYB is abundant in ESCs [27], suggesting that such a compensatory role is unlikely here. Third, failure to completely knockdown a gene product using a knockdown approach would also be expected to differentially affect gene expression due to reduced but not ablated B-MYB binding properties to either promoters or protein complexes that affect individual gene expression. This is possible since the change in target DNA binding activity, as evidenced by ChIP, is incomplete. This latter possibility is of particular interest because the presence of B-MYB in ESCs is rapidly reduced with differentiation, suggesting that decreases in expression may affect fate decisions or the establishment of a definitive cell cycle typical of somatic cells. Fourth, we have also presented data showing that some of the effects reported here may be non-specific and dependent on the shRNA employed. This was not the case for OSN, since shRNA1, 2 and 5 all caused significant decreases in their expression; however, the discrepancies seen with p21^Cip1^ between shRNA1 and shRNA5 suggest that some caution should be taken when interpreting these results. Finally, it is noteworthy that we did not reproduce our previous results regarding B-MYB binding to the *oct4* promoter. We attribute this to the use of different antibodies in the two studies. In fact, the antibody (sc-725) previously employed was found to be unsuitable in ChIP analyses by GenPathway (this study); however, the antibody (sc-724) employed here for ChIP experiments has been successfully utilized in ChIP assays by other groups studying B-MYB [72]. Since we were unable to show B-MYB binding to the *oct4* promoter with sc-724, we conclude that B-MYB does not in fact bind to the *oct4* promoter in mouse. The majority of the data presented in this paper is, however, consistent with both the knockdown and knockout reports for B-MYB in ESCs. Thus we conclude that the bioinformatics analyses and data presented in this paper are valuable and highly indicative of the functional roles of B-MYB in PSCs.

### Summary and conclusions

**Figure 8:**
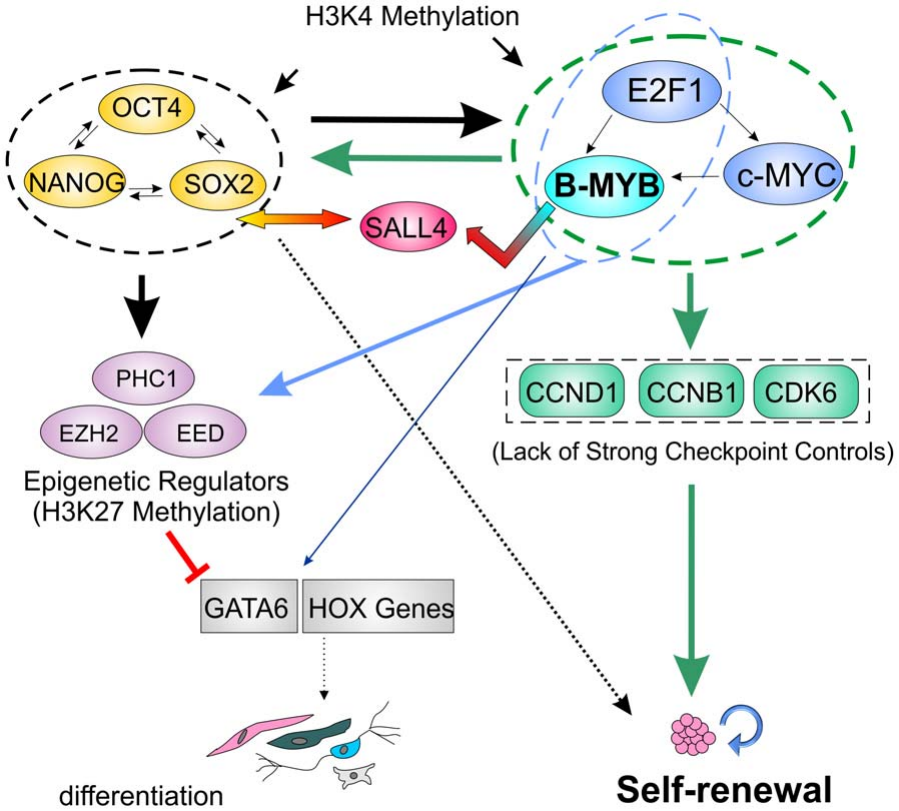
Model depicting transcriptional interactions between pluripotency factors and cell cycle regulators in the maintenance and control of fate decisions that regulate choices between self-renewal and differentiation. In the undifferentiated state, B-MYB promotes high level expression of cell cycle modulators (Green rectangles), pluripotency factors (Yellow ovals) and epigenetic regulators (Purple ovals) to maintain self-renewal; however, upon loss of B-MYB, the subsequent reduction in these factors promotes fate choices that foster differentiation and loss of self-renewal processes. Arrows indicated transcriptional control mechanisms involving epigenetic regulation and/or transcription factor binding, including the postulated indirect effects of B-MYB mediated by SALL4. Colored arrows reflect the transcriptional control of those genes highlighted within ovals (Blue – B-MYB, E2F1; Green – B-MYB, c-MYC, E2F1; Black – Oct4, SOX2, Nanog). GATA6 and HOX genes (Grey rectangles) bind B-MYB, but are transcriptionally inactive unless differentiation is initiated.

B-MYB is present in all mammalian cells in proportion to the degree of cell proliferation. In embryonic carcinoma and somatic cells, B-MYB is required for active transcription of G2/M genes through interactions with the DREAM/LIN Complex [58], and B-Myb depletion in mouse ESCs results in DNA duplication defects in S phase [28], aneuploidy and defects during mitosis [27]. While the importance of B-MYB to cell cycle progression in ESCs has not been disputed, its mechanism of transcriptional control has been, until now, poorly understood in PSCs. In this study, we have highlighted the importance of B-MYB in self-renewal and proposed a model for B-MYB as a key regulator of stem cell and cell cycle genes (Figure 8). The findings presented here indicate that B-Myb is absolutely essential for ESC self-renewal and cell identity through a complex transcriptional network that affects cell cycle regulators, co-expression networks, apoptosis, chromatin and histone modifiers, and transcription factors involved in fate decisions. The self-renewal processes regulated by B-MYB are therefore pivotal for ESC, and by extension iPSC homeostasis. Finally, the results from this principally “omic” study suggest the need for continued experiments designed to functionally assess specific pathways linking B-MYB with self-renewal and differentiation. Of particular interest will be an assessment of when B-MYB binds these target genes during differentiation and cell cycle progression and how their activation may affect fate choices. Other experiments will be required to show how repression of cell cycle progression in somatic cells is overcome through the reprogramming process, and specifically how B-MYB activation and its interactions with the LIN complex foster the generation of PSCs. These studies should lead to a more profound understanding of self-renewal and how reprogramming fosters the establishment of a PSCs that are therapeutically viable.

## Materials and Methods

### ESCs and B-MYB deficient cells

The murine R1 ESC line obtained from A Nagy (Toronto) [73] and the 2D4 iPSC line obtained from K Hochedlinger (Boston) [74] were cultivated on feeder layers of mouse embryonic fibroblasts (MEFs) or under feeder free conditions as described [27], [74], [75]. All experiments with animals used for the preparation of MEFs were in accordance with NIH guidelines under the auspices of the National Institute on Aging Animal Care and Use Committee approved animal study protocol (193-LCS-mi), which was reviewed and approved by the committee on 04/22/2010. The National Institute on Aging animal care & use program is fully accredited by AAALAC International and operates under an Animal Welfare Assurance from the Office of Laboratory Animal Welfare. Plasmid DNA containing shRNA1 (shRNA2 and shRNA5) specific for B-MYB and non-silencing controls were introduced into ESC using the Nucleofector mouse ES Cell Kit from Lonza (Cologne, Germany Cat. No VPH-1001) according to the manufacturer's instructions as previously described [27]. After nucleofection, the cells were plated for 18–24 hours, before the addition of puromycin. Cells were harvested and prepared for microarrays, PCR or ChIP analyses 24 hours after the addition of puromycin (n = 4, biological replicas, for each), unless noted otherwise. Ccnb1 (Accession no. NM_172301) miR target sequences were determined using Invitrogen's BLOCK-iT™ RNAi Designer. Three sequences with start positions in the ORF Region of Ccnb1 mRNA (position 682: GCT ATC CTC ATT GAC TGG CTA; 744: GTA CAT GAC TGT GTC CAT TAT; and 826: GCC ATG TTT ATT GCA AGC AAA) with a predicted high probability of knockdown were selected to create the miR RNAi primers, which were then inserted into the pCDNA6.2-GW/EmGFP-miR expression vector using BLOCK-iT™ Pol II miR RNAi Expression Vector kit with EmGFP (Invitrogen). Plasmid DNA was titrated into ES cells at 5, 10, 15, 20, 30 and 40 µg/2×10^6^ cells using the Nucleofector mouse ES Cell Kit as described, and cells were cultured for 24 hours followed by selection with 60 µg/ml blasticidin for 24 or 48 hours prior to being isolated as previously described [27]. By western blot, the most effective miR construct for Cyclin B1 knockdown was determined to be miR Ccnb1 682 (30 µg) and this miR was used for all further Cyclin B1 knockdown functional analysis.

### Cell Analyses

For determination of colony cell numbers, ESCs were labeled with DAPI and visualized using an EVOS-fl microscope (AMG) and mitotic spindles and centrosomes evaluated as previously described [27]. DNA cell cycle analysis was measured on propidium iodide (PI, 10 µg, Sigma)-stained nuclei using a Becton Dickinson FACs CANTO II. Cell cycle compartments were deconvoluted from single-parameter DNA histograms of 20,000 cells using Multicycler software. 5-Bromodeoxyuridine (BrdU) incorporation was achieved in cells pulsed (45 min) with 10 µM BrdU (Sigma), followed by fixation and incubation with rabbit polyclonal antibodies p-BMyb(Thr490)(sc-20209), p-BMyb(Thr497)(sc-20210) or p-BMyb(Ser581)(sc-20211)(Santa Cruz Biotechnologies) followed by incubated with an Alex Fluor 568 conjugated goat anti-rabbit IgG secondary antibody (Invitrogen). Cells were fixed again and then incubated with an Alexa Fluor 488 conjugated anti-BrdU antibody (Invitrogen), followed by counterstaining with 1 µg/ml Hoechst 33342 or TOPRO-3. Imaging was with either a Zeiss LSM-*310* Laser Scanning *Confocal* Microscope or inverted Zeiss microscope with SPOT camera imaging system. For flow analyses, ESCs were dissociated with trypsin and fixed in methanol/acetone (1∶1) prior to incubation with rabbit polyclonal antibodies to phosphorylated forms of B-MYB. Cells were incubated in Alexa Fluor 488 conjugated goat anti-rabbit IgG (Invitrogen) and counterstained with 10 µg/ml PI containing 1 mg/ml RNAse A prior to flow cytometry as described [27].

### ChIP experiments and data analysis

Chromatin was prepared from ESCs nucleofected with a non-silencing control or shRNA1 to B-MYB as described 48 hours after nucleofection and including 24 hours of puromycin selection [27]. Five commercially available antibodies (Santa Cruz: sc-724, sc-725, sc-13028, sc-81192; Abcam: ab12296) were tested for their ability to pull-down B-MYB bound chromatin of two known target genes: cell division cycle-associated protein 2 (Cdca2) and Cyclin B1 (Ccnb1). Only one antibody (sc-724) effectively precipitated chromatin regions containing Cdca2 and Ccnb1 above the negative control sequence threshold (Untr6) and isotype controls. Using this antibody, three independent assays from R1 ESCs and two additional samples from ESCs nucleofected with a shRNA against B-MYB or non-silencing control and selected with puromycin were analyzed. For each experiment 5×10^6^–1×10^7^ cells were fixed by the addition of one-tenth volume of formaldehyde solution (11% Formaldehyde, 0.1 M NaCl, 1 mM EDTA pH 8.0, 50 mM HEPES pH 7.9) to the cultivation media, followed by agitation for 15 minutes at room temperature. Fixation was stopped by the addition of one twentieth volume 2.5 M glycine for 5 minutes. Cells were then transferred to conical tubes and kept at 4°C for the remainder of the procedure. After cooling, cells were centrifuged at 800 g for 10 minutes, and then re-suspended in 10 mL chilled PBS-lgepal solution (1× PBS, 0.5% lgepal CA-630 (Sigma #I-8896)) and triturated. Following another centrifugation, 10 mL of chilled PBS-lgepal was added to each tube, followed by the addition of PMSF in ethanol (Sigma P-7626) to a final concentration of 1 mM. The cells were centrifuged again, the supernatant removed, and the cell pellets snap frozen on dry ice and stored at −80°C. All samples with appropriate controls were shipped to GenPathway Inc (San Diego, CA) who finished processing the samples for ChIP. Hybridizations were performed using GeneChip Mouse Promoter 1.0R Arrays (Affymetrix), which contained information from ∼8 kb upstream and 2 kb downstream of 25,500 transcription start sites of known mouse promoter regions.

Briefly, data were generated by GenPathway using a standard curve of genomic DNA and known copy numbers without normalization for primer pair efficiency. The binding events per 1000 cells for each genomic region were calculated from an average of triplicate qPCR runs for each test, and data were normalized to sequences with no binding to B-MYB (i.e., Untr regions). Genpathway indicated that changes in factor binding as low as 1.3× could be reproducibly demonstrated. In these experiments, only signals with a threshold of 2.2 or greater than regions not bound by B-MYB were considered positive. Data reported here were combined from control nucleofections and the three mouse R1 ESC samples. BED files were analyzed using the UCSC genome browser and tiling analysis software available from Affymetrix. Confirmatory CHiP assays were performed as previously described [27]. Cis-elements were analyzed using bioinformatics tools available from Genomatix, Inc (http://www.genomatix.de/en/index.html, Munich, Germany). Functional Annotations and Functional Classes (High and Medium Stringency) were determined with the Database for Annotation, Visualization and Integrated Discovery (DAVID) v6.7 (http://david.abcc.ncifcrf.gov/) and Ingenuity Systems IPA (Redwood City, California).

Independent ChIP analyses employing sc-724 were performed to confirm the ChIP-chip results acquired by GenPathway. Antibodies to rabbit IgG (Santa Cruz, Cat No. sc-2027) were employed as a negative control for immunoprecipitation. For data analyses, we used a fold enrichment protocol described at (http://www.invitrogen.com/etc/medialib/files/Cell-Culture/XLSs.Par.85244.File.dat/ChIP_Data_Analysis_Template.xls). In contrast to the GenPathway protocol, data were normalized relative to IgG immunoprecipitations and total DNA input as previously described [27]. All normalizations were defined in step 2 of the web link listed above, where IgG fold enrichment equals 1. Through these independent experiments, we were able to confirm binding of B-MYB to promoter regions for multiple gene targets that were originally identified by ChIP-chip.

### Microarray experiments and data analysis

RNA was prepared from ESCs nucleofected with a non-silencing control or shRNA to B-MYB as described 48 hours after nucleofection and including 24 hours of puromycin selection [27]. All subsequent reactions were performed in the NIA Gene Expression and Genomics Unit and normalized as previously described [76]. Total RNA was used to generate biotin-labeled cRNA using the Illumina TotalPrep RNA Amplification Kit. Double stranded cDNA was incubated and transcribed overnight to generate single-stranded RNA (cRNA) containing biotin-16-UTP. A total of 0.75 µg of biotin-labeled cRNA was hybridized at 58°C for 16 hours to Illumina's Sentrix Mouse Ref-8 Expression BeadChips (Illumina, San Diego, CA). Each BeadChip has ∼25,600 well-annotated RefSeq transcripts with approximately 30-fold redundancy. Labeled cRNA was detected by staining with streptavidin-Cy3. Hybridized arrays were scanned using an Illumina BeadStation 500× Genetic Analysis Systems scanner and the image data extracted using the Illumina GenomeStudio software, version 1.1.1.1. Data will be deposited to Gene Expression Omnibus (GEO) upon manuscript acceptance. The microarray gene expression data were normalized using the quantile method. Normalized data were further converted into log2 ratios of expression values over the average expression value across all the samples for each probe. The genes differentially expressed between normal and B-MYB knockdown cells were identified by a t-test, with the P value adjusted for the false discovery rate using the Benjamini-Hochberg algorithm. The fold-change of the gene expression level was measured as the difference of mean expression levels between control and B-MYB knockdown cells. Significantly enriched biological processes and pathways were identified through GSEA analysis [36] and Fisher's exact tests [77], based on Gene Ontology (GO) and KEGG database. In this study, we report GO chart specificity levels 2 and 3 for biological processes. The significantly enriched regulatory factors were identified in the same ways, based on the database provided by the GSEA website (http://www.broadinstitute.org/gsea/msigdb/). Gene names, unless otherwise indicated, are in accordance with standard Mouse Genome Informatics Nomenclature: http://www.informatics.jax.org/. Genes are listed in italics, transcripts with the first letter capitalized and proteins in fully capitalized letters.

### qPCR analyses

ES R1 cells were passaged off of feeder layers, and three independent samples were prepared for each experimental conditional. 2×10^6^ cells were nucleofected with 15 µg of appropriate plasmid (pSuper, non-silencing (NS) shRNA shRNA1, shRNA2, shRNA5) as previously described [27]. Briefly, plasmid DNA was introduced into ESCs using the Nucleofector mouse ES Cell Kit from Amaxa Biosystems (Cologne, Germany Cat. No VPH-1001) according to the manufacturer's instructions. Cells were harvested for RNA extraction at 48 and 72 hours after nucleofection (i.e., 24 and 48 hours after selection with 1 µg/ml of puromycin (Sigma)). RNA was extracted with the RNA mini Kit (Quiagen), and qPCR was performed with SybrGreen Kit (Invitrogen) according to the manufacturer's instructions in duplicate or triplicate. Primers employed in this study are shown in Table 5 or were previously reported [27].

**Table 5:** Primers used for QPCR detection.

Name	Official name	Fw	Rv	bp
Ink4b	Cdkn2b	CGCTGCCACTGGAGATTGA	TCGTGCTTGCAGTCTTCCTAGA	90
Cip1	Cdkn1a	CAGGCACCATGTCCAATCCT	GAGACAACGGCACACTTTGCT	68
GADD45	GADD45	TGCAGAGCAGAAGACCGAAA	ACCGTAATGGTGCGCTGACT	90
Skp2	Skp2	TCAGTGGACACCATGCATAGGA	ATCCCATCCCCACGTGAA	87
p107	Rbl1	GCAGATGATGACTATTGCCAAAGA	GGGTCTGCCCTGAAATGTACTT	84
E2F1	E2F1	CTGGACCACCTGATGAATATCTGTAC	CAATGCTACGAAGGTCCTGACA	106
DP-1	Tfdp1	TCTGCCAGTGATTTGAGCAATG	GACCCTGGAGCCGCTGTA	81
Dbf4	Dbf4	GCTTCAGAGCCCACAACCTATT	GGTCAGGCTCACTTGCATTGA	88
Bub1	Bub1	CAGCATCTTTACCCTGTCCTAGTCA	CCACTGTCGCATGGTCAATG	111
Phc1	Phc1	CCATCCACGCCAGAGTTACA	TCCTCGACGCTCCATTGG	74
Ezh2	Ezh2	CAAATCTGTTCAGAGGGAGCAAA	CACTTACGATGTAGGAAGCAGTCATACT	93
EED	EED	ATGCACAACACTGACCCATCA	GATGCTGCTATCCCTACTGAAACTG	73
Cdc20	Cdc20	ACCTGGAGGTGACCGCTTTA	CGGCTGGTTTTCCTTGCTT	85
Plk1	Plk1	AGCCGGCGGCAGTATGTA	CTTTTGTGTCTGCGTCTGAGATCT	85
CDK2	CDK2	CCATTCTCACCGTGTCCTTCA	AAAGTCTGCCAGCTTGATGGA	85
CycA	Ccna2	TGTGAAGATGCCCTGGCTTT	TCAAAACTGCCATCCATTGG	95

### Comparative analyses

For more comprehensive analyses, ChIP-seq and ChIP-chip tiling array data from mouse ES cells for various TFs and histone methylations were downloaded, and candidate genes whose promoters were occupied by these regulators identified. The ChIP-seq data (bed file) were downloaded from the GEO database of NCBI: GSE1224 for the data contained H3K4me3 and H3K27me3; GSE 11431 for the data contained 13 TFs (Nanog, Oct4, STAT3, Smad1, Sox2, Zfx, c-Myc, n-Myc, Klf4, Esrrb, Tcfcp2l1, E2f1 and CTCF) and two transcription regulators p300 and Suz12. These datasets were generated from the mouse ES cell line V6.5 [47] and E14 [44], respectively. Further binding targets of TFs (Nanog, Oct4, Sox2, c-Myc, Tcf3, Klf4, Dax1, Rex1, Dpf281 and Nac1) and histone methylation (H3K4me3) were obtained from ChIP-seq experiments on mouse ES cells V6.5 [47] and from ChIP tiling array experiments on the mouse ES cell line J1 [48]. Specifically comparisons were performed with ChIP-seq data of mouse ES cells V 6.5 to evaluate histone methylation patterns [40], [47].

ChIP-seq data in bed format were analyzed by the cisGenome software (http://www.biostat.jhsph.edu/~hji/cisgenome/index.htm). The bed data contain chromosome locations for all binding sequence reads of TFs or histone methylations. We first mapped the reads or tags of each dataset to the mouse genome (version mm8). We then measured whether there were binding tags located at the promoter region within 1000 bp of both upstream and downstream from the transcriptional start site. Putative binding targets were defined as promoters bound by TFs and histone methylation marks were also evaluated. We subsequently identified the candidate target genes of each TF and histone methylation in mouse ES cells.

### Identification of co-expression gene clusters within pathways

Conserved and divergent co-expression patterns were identified from gene expression data of ES cells under control and B-MYB knockdown conditions, respectively, using a comparative clustering method [65]. Pathway data were adopted from the KEGG database (www.genome.ad.jp/kegg). Although a total of 51 pathways showed significant associations with B-MYB, we report only two gene co-expression patterns essentials to ES cell signaling pathways: cell cycle and apoptosis.

### Construction of global gene co-expression networks

Global co-expression networks of ESCs were constructed from microarray data derived under both control and knockdown conditions. The Pearson correlation coefficient value (*r*) was first calculated based on the expression profiles for each gene pair. The co-expression links in the network were kept if the corresponding *r* values are at or above a threshold. The threshold value was determined according to the scale-free criterion, which is measured by the square of the correlation coefficient (*R*) between *log(P(k))* and *log (k)*, where *k* denotes the connectivity of a node, or the number of links of a node to other nodes in a network. *P(k)* gives the probability that a selected node has exactly *k* links, which is calculated as the number of the nodes (genes) at a given *k* value divided by the total number of nodes. The *R* value of 0.80 corresponded to *p*<0.03, so that was selected as the threshold value. At or above the threshold value, genes were considered to be co-expressed and the derived networks obeyed a power law distribution and were scale-free. Such a scale-free criterion removed possible spurious co-expression links and so that the resulting networks are biologically meaningful.

### Statistics

Cell data are presented as mean ± S.E.M., unless indicated otherwise, and statistical analyses between groups were performed with an unpaired T test. The statistical significance of overlaps between target gene sets of different TFs was assessed using a Fisher's exact test according to the hypergeometric distribution. To perform the Fisher's exact test, we calculated the enrichment level *R* of two gene set overlaps based on an hypergeometric distribution as described in [54]. Specifically, given a total of *N* genes, if gene sets A and B contain *m* and *n* genes, respectively, and *k* of them are in common, then the *P* value of enrichment is calculated by:
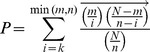
The enrichment level of the overlap is calculated by *R = kN/(mn)*. *P* values less than 0.05 were considered significant.

## Supporting Information

Table S1 **Differentially expressed genes (fold-change ≥1.5) identified from microarray experiments, their overlaps with target genes of TFs, and histone modifications in mouse ESCs.** The differentially under- or over-expressed genes with the fold-change ≥1.5 were identified in mouse ES cells R1 by B-Myb knockdown-dependent microarray experiment. Target genes of B-MYB were identified by ChIP-chip in ES cells R1. Target genes of TFs and histone modifications were identified by ChIP-seq or ChIP tilling array on mouse ES cells E14, J1 and V 6.5 (see methods for details).
(XLS)

Table S2 **GSEA to identify pathways that significantly differ between control and knockdown conditions.** A) Enriched KEGG pathways in response to B-MYB knockdown, identified by using GSEA, based on 18,097 expressed genes in ESCs. In this analysis, a total of 18,097 genes were ranked by fold change of gene expression between the control and B-MYB knockdown in mouse ES cells R1 (see Materials and Methods). The ranked genes were then analyzed by GSEA based on Kegg pathways. The pathways in the table represents enriched ones for under-expressed gene set after B-MYB knockdown and with GSEA p value <0.05 and FDR q value <0.25. B) Most significant biological processes associated with B-MYB knockdown induced over-expressed genes. For these analyses, a total 18,097 genes were ranked by fold change of gene expression between control and BMYB knockdown in mouse ESCs based on microarray experiments (see Methods). Ranked genes were analyzed using GSEA based on GO biological processes. The biological processes in the table represents enriched ones with P value of 0 for over-expressed genes by B-MYB knockdown.
(XLS)

Table S3 **Enriched transcription factors in response to B-MYB knockdown in ESCs identified by GSEA.** A total 18,097 genes were ranked by fold-change of gene expression between the control and B-MYB knockdown in mouse ES cells R1 by microarray experiments (see materials and Methods). The ranked genes were then analyzed by GSEA based on KEGG pathways. The pathways in the table represents enriched ones for under-expressed gene set after B-MYB knockdown and with GSEA p value <0.05 and FDR q value <0.25.
(XLS)

Table S4 **Co-expression gene clustering in two ESC-critical pathways.** A) Cell cycle pathway. B) Apoptosis pathway.
(XLS)

Table S5 **Global co-expression networks in ESCs under control and B-MYB deficiency conditions.** A) Genes showing high connectivity in the co-expression network of the control ESCs. B) Genes showing high connectivity in the co-expression network of B-MYB knockdown ESCs. C) Most prevalent genes with differential connectivity in the co-expression networks of the control ESCs. D) Most prevalent genes with differential connectivity in the co-expression networks of B-MYB knockdown ESCs. Target genes of Oct4, Sox2, Nanog, c-Myc and E2f1 were identified by ChIP-seq or ChIP tilling array on mouse ES cells E14, J1 and V 6.5 (see Materials and Methods). 1-3O, 1-3S, 1-3N and 1-2M represent target gene of Oct4, Sox2, Nanog and c-Myc identified at 1-3 ES cell, respectively. B-MYB target genes were identified by ChIP-chip. KD – knockdown.
(XLS)

Table S6 **ChIP-chip datasets derived from hybridizations to Affymetrix GeneChip Mouse Promoter 1.0R Arrays.**
(XLS)

Table S7 Functional annotations of ChIP-chip determined B-MyB target genes based on Fisher's exact tests and GO terms: A) functional classes with high stringency; B) functional classes – gene list with high stringency; C) functional classes with medium stringency; D) functional classes – gene list with medium stringency.
(XLS)

Table S8 *Cis*-element analysis of the 1020 gene promoters that bound B-MYB and predicted frequency of these elements in genomic and promoter regions.
(XLS)

Table S9 Overlaps between the 775 informative target genes of B-MYB and target genes of Oct4, Sox2, Nanog, H3K4me3, and H3K27me3. All 775 genes in the table are B-MYB target genes identified by ChIP-chip and overlapped with B-MYB knockdown microarray dataset (18,097 genes) in mouse ES cells R1. Target genes of Oct4, Sox2, Nanog, H3K4me3, and H3K27me3 were identified by ChIP-seq or ChIP-chip tilling array on mouse ES cells E14, J1 and V 6.5 (see Materials and Methods).
(XLS)

Table S10 Pathway-specific genes bound by B-MYB, Oct4, Sox2, Nanog, c-Myc and E2f1 in mouse ESCs. 1-2O, 1-2S, 1-2N and 1-2M represent target genes of Oct4, Sox2, Nanog and c-Myc identified in one and two mouse ES cells, respectively. Target genes of Oct4, Sox2, Nanog, c-Myc, E2f1, H3K4me3, and H3K27me3 were identified by ChIP-seq or ChIP tilling array on mouse ES cells E14, J1 and V 6.5 (see Materials and Methods). 1O, 1S, 1N and 1M represent target gene of Oct4, Sox2, Nanog and c-Myc identified at one ES cell, respectively. 2O, 2S, and 2N represent target gene of Oct4, Sox2, Nanog and c-Myc identified at 2 ES cells, B-MYB target genes were identified by ChIP-chip.
(XLS)

Figure S1 Distribution of connections per node in the co-expression networks. Nodes are genes and connections are defined by co-expression between two genes. We calculated the correlation (r) between each gene according to its expression profile, and determined links on the network if r was above the threshold value. Co-expression was defined by a correlation in expression profiles (r) higher than 0.75, 0.80, 0.85, and 0.90, with p-value of 0.03, respectively. The distributions at r≥0.90 showed non-random scale-free topology. The X-axis shows the number of connections (K), and Y-axis shows the number of nodes [N] that have the corresponding number of connections in the networks of normal and B-MYB knockdown cells. The numbers are shown on the log_10_ scale.
(TIF)
