# Supplementary figures and images for: The B-MYB Transcriptional Network Guides Cell Cycle Progression and Fate Decisions to Sustain Self-Renewal and the Identity of Pluripotent Stem Cells
Source: PLoS One. 2012 Aug 24;7(8):e42350. doi: 10.1371/journal.pone.0042350 (PMC3427317; doi:10.1371/journal.pone.0042350)

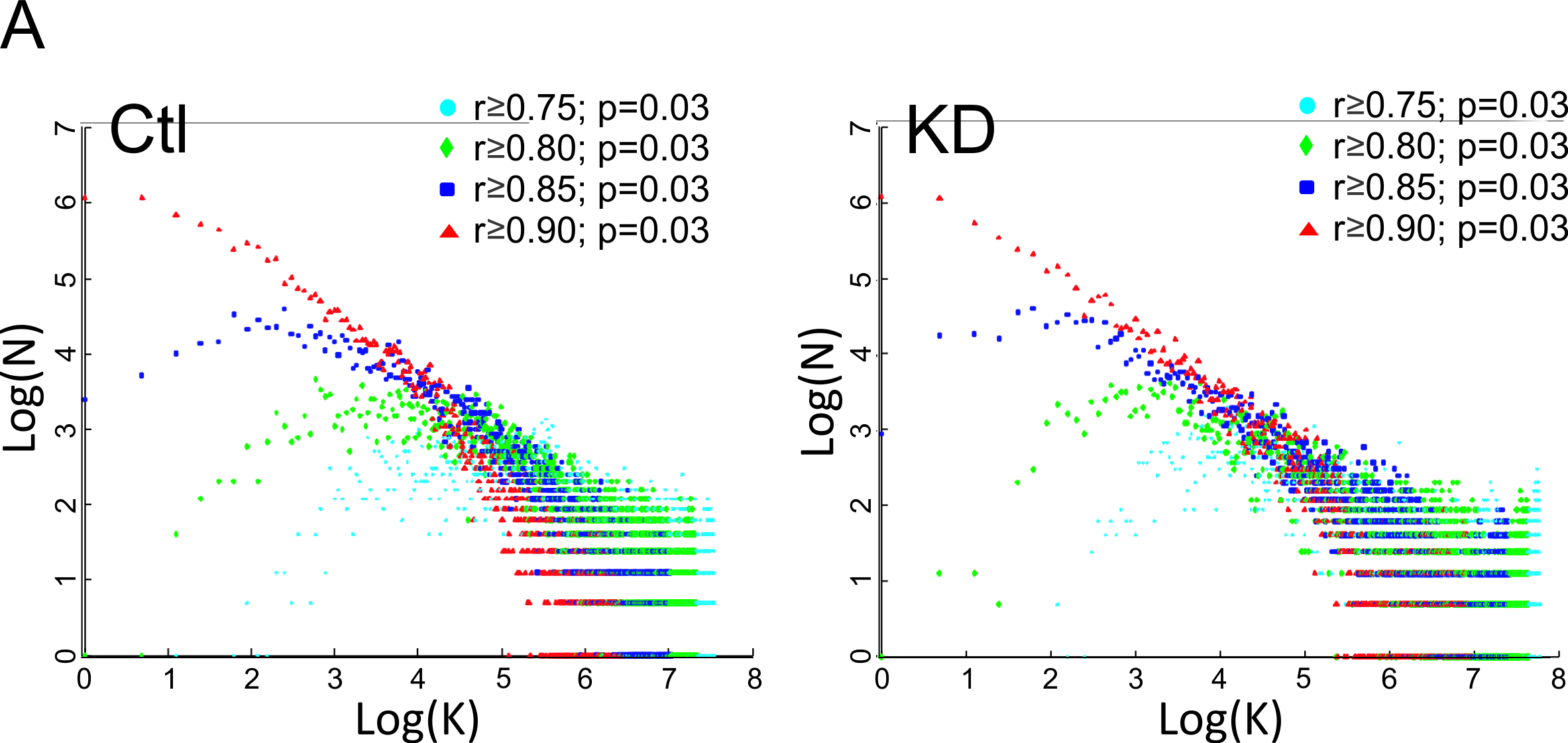

Supplement: Figure S1 — Distribution of connections per node in the co-expression networks. Nodes are genes and connections are defined by co-expression between two genes. We calculated the correlation (r) between each gene according to its expression profile, and determined links on the network if r was above the threshold value. Co-expression was defined by a correlation in expression profiles (r) higher than 0.75, 0.80, 0.85, and 0.90, with p-value of 0.03, respectively. The distributions at r≥0.90 showed non-random scale-free topology. The X-axis shows the number of connections (K), and Y-axis shows the number of nodes [N] that have the corresponding number of connections in the networks of normal and B-MYB knockdown cells. The numbers are shown on the log10 scale. (TIF) [file pone.0042350.s011.tif]
